# Nitrogen isotope evidence for Earth’s heterogeneous accretion of volatiles

**DOI:** 10.1038/s41467-022-32516-5

**Published:** 2022-08-15

**Authors:** Lanlan Shi, Wenhua Lu, Takanori Kagoshima, Yuji Sano, Zenghao Gao, Zhixue Du, Yun Liu, Yingwei Fei, Yuan Li

**Affiliations:** 1 State Key Laboratory of Isotope Geochemistry, Guangzhou Institute of Geochemistry, Chinese Academy of Sciences, Guangzhou, 510640 China; 2 CAS Center for Excellence in Deep Earth Science, Guangzhou, 510640 China; 3 College of Earth and Planetary Sciences, University of Chinese Academy of Sciences, Beijing, 100049 China; 4 Division of Ocean-Earth System Science, Atmosphere and Ocean Research Institute, University of Tokyo, Kashiwa, Chiba 277-8564 Japan; 5 Center for Advanced Marine Core Research, Kochi University, Nanokoku, Kochi 783-8502 Japan; 6 International Center for Planetary Science, College of Earth Sciences, Chengdu University of Technology, Chengdu, 610059 China; 7 Earth and Planets Laboratory, Carnegie Institution for Science, Washington DC, 20015 USA

**Keywords:** Geochemistry, Core processes

## Abstract

The origin of major volatiles nitrogen, carbon, hydrogen, and sulfur in planets is critical for understanding planetary accretion, differentiation, and habitability. However, the detailed process for the origin of Earth’s major volatiles remains unresolved. Nitrogen shows large isotopic fractionations among geochemical and cosmochemical reservoirs, which could be used to place tight constraints on Earth’s volatile accretion process. Here we experimentally determine N-partitioning and -isotopic fractionation between planetary cores and silicate mantles. We show that the core/mantle N-isotopic fractionation factors, ranging from −4‰ to +10‰, are strongly controlled by oxygen fugacity, and the core/mantle N-partitioning is a multi-function of oxygen fugacity, temperature, pressure, and compositions of the core and mantle. After applying N-partitioning and -isotopic fractionation in a planetary accretion and core–mantle differentiation model, we find that the N-budget and -isotopic composition of Earth’s crust plus atmosphere, silicate mantle, and the mantle source of oceanic island basalts are best explained by Earth’s early accretion of enstatite chondrite-like impactors, followed by accretion of increasingly oxidized impactors and minimal CI chondrite-like materials before and during the Moon-forming giant impact. Such a heterogeneous accretion process can also explain the carbon–hydrogen–sulfur budget in the bulk silicate Earth. The Earth may thus have acquired its major volatile inventory heterogeneously during the main accretion phase.

## Abstract (web summary)

How and when Earth acquired its major volatiles N-C-H-S remains unclear. Here the authors show that Earth may have acquired its major volatiles from both reduced and oxidized impactors before and during the Moon-forming giant impact.

## Introduction

Both dynamic models^1,2^ and observational evidence^3,4^ indicate delivery of volatiles by volatile-rich asteroids to the inner solar system; however, the mechanism for the accretion of Earth’s major volatiles (N–C–H–S) remains unresolved^5–8^. Some argued that Earth accreted its volatiles from carbonaceous chondrite (CI type)-like materials in the form of undifferentiated “late veneer” after core-formation ceased^9,10^, as evidenced by the CI chondrite-like S, Se, and Te ratios, and Se isotopes, in the bulk silicate Earth (BSE)^11,12^. However, some argued that Earth accreted its volatiles from oxidized chondritic materials at its full or late accretion stages, in which volatiles participated in Earth’s core-formation in the magma ocean^2,13–16^. Some models also proposed that Earth acquired its volatiles from a single giant impactor, such as the Moon-forming impactor^17–19^. Although some^7,16–18^ of these previous models have attempted to explain the major volatile budget and ratios in the BSE, it remains unknown whether such models are consistent with the observed isotopes of major volatiles in the BSE.

**Fig. 1:**
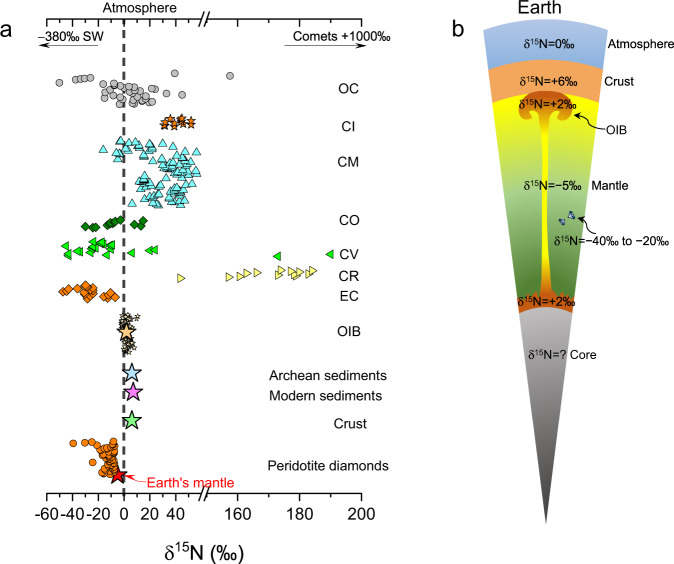
The N-isotopic composition (δ^15^N) of cosmochemical and geochemical reservoirs. **a**, **b** Earth’s mantle δ^15^N, inferred from fibrous diamonds and mid-ocean ridge basalts, converges towards a globally uniform value of −5‰; however, in some mantle peridotite diamonds, the δ^15^N are as low as −40‰, which are comparable to EC δ^15^N. The positive OIB δ^15^N are regarded to be primordial features of Earth’s deep mantle^30^. Earth’s sediments and crust also show positive δ^15^N. Solar wind (SW) and comets show extremely negative and positive δ^15^N, respectively. Note that Earth’s atmosphere δ^15^N is zero. All δ^15^N data were taken from references compiled in Supplementary Note 1.

The N-isotopes (^14^N and ^15^N) are excellent proxies in tracing the sources of volatiles accreted to the terrestrial planets^4,5,20,21^ due to their large fractionations among geochemical and cosmochemical reservoirs as summarized in Fig. 1. The δ^15^N values of Earth’s mantle (δ^15^N = [(^15^N/^14^N)_sample_/(^15^N/^14^N)_standard_ – 1)] × 1000, where the standard is the atmospheric N_2_), inferred from fibrous diamonds and mid-ocean ridge basalts, are mainly between −10‰ and 0‰ and converge towards a globally uniform value of −5‰ (ref. 22). More negative δ^15^N values down to −20‰ and −40‰ were observed in diamonds from Earth’s deep mantle, which were interpreted to be relict primordial N and used to argue for an enstatite chondrite (EC)-origin of Earth’s N^23–25^, because the EC δ^15^N are −45‰ to −15‰ (ref. 26). The average δ^15^N of Earth’s surface (crust + atmosphere) is approximately +3‰ (refs. 22,27), and the δ^15^N imbalance between Earth’s mantle and surface forms a long-standing unresolved puzzle^22^. The δ^15^N of oceanic island basalts (OIB) are overall positive (−2‰ to +6‰), which was usually interpreted as resulting from recycled sediments in the OIB mantle source^28,29^. However, such positive δ^15^N are more likely primordial features because of the inefficiency of deep N-subduction^30^. Most diamond populations of Archean ages also define a mantle δ^15^N of −5‰ (ref. 31); accordingly, the N-isotopic signature of Earth’s different reservoirs may have been established before Archean.

The protosolar nebula (PSN) has δ^15^N as low as −380‰, while comets have δ^15^N up to +1000‰ (Fig. 1). Both cannot be a substantial source of Earth’s volatiles^4,5^, in light of Earth’s mantle δ^15^N of −5‰. The CI and CM chondrite-like materials, which are likely partial sources of Earth’s water^4,5^, have average δ^15^N of +42 to +175‰ (ref. 4). These δ^15^N also exclude CI and CM chondrite-like materials as Earth’s solo N-source. An EC-origin of Earth’s N is essentially consistent with the observation that the BSE and ECs have largely identical isotopic compositions for O, Ca, Ti, Cr, Ni, Mo, and Ru^32,33^. However, if EC-like materials are Earth’s main N-source, it remains unclear how Earth’s N-isotopes have evolved from initial EC δ^15^N to the present-day observations (Fig. 1). The addition of CI chondritic late veneer to the proto-Earth has long been proposed^34^ to cause an increment of Earth’s mantle δ^15^N to −5‰, but Earth’s mantle Ru-isotopes rule out an outer solar system origin of the late veneer^32,33^. The non-chondritic relative volatile abundance in the BSE^5,6^ is also against the late veneer as an important source of Earth’s major volatiles. Two recent studies emphasized that Earth’s mantle δ^15^N could have been achieved from EC-like materials through Earth’s core–mantle segregation and preferential partitioning of ^14^N into Earth’s core^35,36^. If correct, the Earth must have acquired its N and other major volatiles from EC-like materials, rather than from volatile-rich CI or CM chondrite-like materials.

Here we report experimental determinations of N-partitioning and -isotopic fractionation between Fe-rich metallic and silicate melts. Using the newly measured N-partitioning and -isotopic fractionation, we demonstrate that core-formation cannot cause significant core/mantle N-isotopic fractionation, but Earth’s heterogeneous accretion of volatiles before the cessation of core-formation can explain the N-budget and -isotopic composition of Earth’s different reservoirs, which places tight constraints on the accretion process of Earth’s major volatiles.

## Results and discussion

### Metal/silicate N-partitioning

The experiments were performed at 1700–2200 °C, 1–8 GPa, and oxygen fugacity (*f*O_2_) of 0.3–4.7 log units below the iron-wüstite (IW) buffer to determine N-partitioning and -isotopic fractionation between Fe-rich metallic and silicate melts (${D}_{N}^{{{{{{\rm{metal}}}}}}/{{{{{\rm{silicate}}}}}}}$ and ∆^15^ *N*^metal-silicate^), using piston cylinder and multi-anvil devices (Supplementary Data 1, 2 and Methods). The silicate melt NBO/T (the ratio of non-bridging oxygens to tetrahedral cations) was 0.2–3.1. The Fe-rich metallic melts contained 87.0–98.8 wt.% Fe and up to 6.8 wt.% Si, 1.8 wt.% S, and 11.5 wt.% C. The N-content and δ^15^N of quenched samples (Supplementary Fig. 1) were quantified by using a modified noble gas mass spectrometry (Methods). The metallic and silicate melts contained 43–6390 ppm and 44–1590 ppm N (by weight), respectively (Supplementary Data 3, 4). In two N_2_-saturated experiments (LY24 and LY25; Supplementary Data 2), the metallic melts contained ~1.1 and 1.4 wt.% N, respectively, which are comparable to previously determined N-solubility values^37^. Raman spectroscopy measured N–C–H–O species in the quenched silicate melts at *f*O_2_ of ~IW−0.5 included OH^−^, CH_4_/CH, N_2_, NH_3_/NH, and/or H_2_ (Supplementary Fig. 2), consistent with the previous measurements^36,38,39^.

**Fig. 2:**
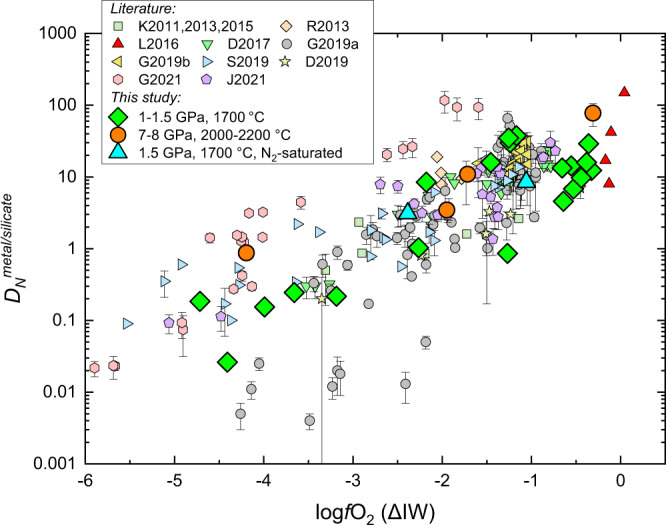
Metal/silicate N partition coefficients (*D*_*N*_^metal/silicate^) as a function of oxygen fugacity. All ${D}_{N}^{{{{{{\rm{metal}}}}}}/{{{{{\rm{silicate}}}}}}}$ data were obtained under largely variable conditions (*P* = 0.85–26 GPa; *T* = 1523–3400 K; *NBO*/*T* = 0.02–3.1; and *f*O_2_ = IW–0.1 to IW–5.5) and were taken from K2011, 2013, 2015 (refs. 64,92,93), R2013 (ref. 94), L2016 (ref. 35), D2017 (ref. 95), G2019a (ref. 40), G2019b (ref. 18), S2019 (ref. 41), D2019 (ref. 36), G2021 (ref. 42), and J2021 (ref. 43). The ${D}_{N}^{{{{{{\rm{metal}}}}}}/{{{{{\rm{silicate}}}}}}}$ are dominantly controlled by *f*O_2_, and the scatter of ${D}_{N}^{{{{{{\rm{metal}}}}}}/{{{{{\rm{silicate}}}}}}}$ at a given *f*O_2_ is caused by the variation of other parameters, such as *P*–*T* and the compositions of metallic and silicate melts as summarized in Eq. (1). Note that all literature *f*O_2_ values were recalculated by using the approach described in Methods. Source data are provided in Supplementary Data 2.

The calculated ${D}_{N}^{{{{{{\rm{metal}}}}}}/{{{{{\rm{silicate}}}}}}}$ varied from 0.03 to 78 (Fig. 2), which are mainly controlled by *f*O_2_ as recognized previously^35,36,40,41^. Note that the present ${D}_{N}^{{{{{{\rm{metal}}}}}}/{{{{{\rm{silicate}}}}}}}$ were determined by using ppm level N in the samples, differing from previous studies (Fig. 2) that added wt.% level N in the samples to measure N by using electron microprobe. The change of N-species in silicate melt as a function of *f*O_2_ has been proposed to explain the ${D}_{N}^{{{{{{\rm{metal}}}}}}/{{{{{\rm{silicate}}}}}}}$–*f*O_2_ correlation^35,36,40^. In oxidized silicate melt (*f*O_2_ > IW), N dissolves physically and mainly as N_2_; however, the other N-species such as CN^-^, NH_3_ and/or N^3-^ dissolve chemically in reduced silicate melt and become dominant at *f*O_2_ <IW–2 (refs. 38,39). Therefore, ${D}_{N}^{{{{{{\rm{metal}}}}}}/{{{{{\rm{silicate}}}}}}}$ decrease with decreasing *f*O_2_ under otherwise equivalent conditions. In addition to *f*O_2_, other parameters such as *P*–*T* and the compositions of metallic and silicate melts also affect ${D}_{N}^{{{{{{\rm{metal}}}}}}/{{{{{\rm{silicate}}}}}}}$ (refs. 40–43). Using the present and previous ${D}_{N}^{{{{{{\rm{metal}}}}}}/{{{{{\rm{silicate}}}}}}}$ (Fig. 2) and the multiple linear regression approach, we derived a comprehensive equation to describe ${D}_{N}^{{{{{{\rm{metal}}}}}}/{{{{{\rm{silicate}}}}}}}$:

1 $${{\log }}{D}_{N}^{{{{{{\rm{metal}}}}}}/{{{{{\rm{silicate}}}}}}} =	 -0.38(0.34)+\frac{3370\left(522\right)}{T}+75(32){{\cdot }}\frac{P}{T}\\ 	+0.56(0.06)\cdot {NBO}/T+0.47(0.03)\cdot \triangle {IW}\\ 	+1.97(0.55)\cdot {{\log }}(1-{x}_{S}^{{{{{{\rm{metal}}}}}}})+5.73(0.75)\cdot {{\log }}(1-{x}_{C}^{{{{{{\rm{metal}}}}}}})\\ 	+3.88(1.37)\cdot {{\log }}(1-{x}_{{Ni}}^{{{{{\rm{metal}}}}}})+5.52(1.17)\cdot {{\log }}(1-{x}_{{Si}}^{{{{{{\rm{metal}}}}}}})({{{\rm{R}}}}^2=0.81{{;}}{{\sigma }}=0.47)$$

where *T* is temperature in K, *P* is pressure in GPa, ΔIW denotes *f*O_2_ relative to the IW buffer, and ${x}_{i}^{{{{{{\rm{metal}}}}}}}$ is the mole fraction of element *i* in the metallic melt. The experimental data cover pressures from 0.85 to 26 GPa, temperatures from 1523 to 3400 K, silicate melt NBO/T from 0.02 to 3.12, and *f*O_2_ from IW–0.1 to IW–5.9 (*n* = 241). The agreement within 0.5 log units between the experimentally determined ${{\log }}{D}_{N}^{{{{{{\rm{metal}}}}}}/{{{{{\rm{silicate}}}}}}}$ and the predicted values using Eq. (1) indicates the consistence of our new ${D}_{N}^{{{{{{\rm{metal}}}}}}/{{{{{\rm{silicate}}}}}}}$ and previous data, including those obtained under C-undersaturated conditions (Supplementary Fig. 3). Equation (1) indicates that increasing pressure or NBO/T would increase ${D}_{N}^{{{{{{\rm{metal}}}}}}/{{{{{\rm{silicate}}}}}}}$, but increasing temperature or the light element content in metallic melt would decrease ${D}_{N}^{{{{{{\rm{metal}}}}}}/{{{{{\rm{silicate}}}}}}}$.

### Metal/silicate N-isotopic fractionation

**Fig. 3:**
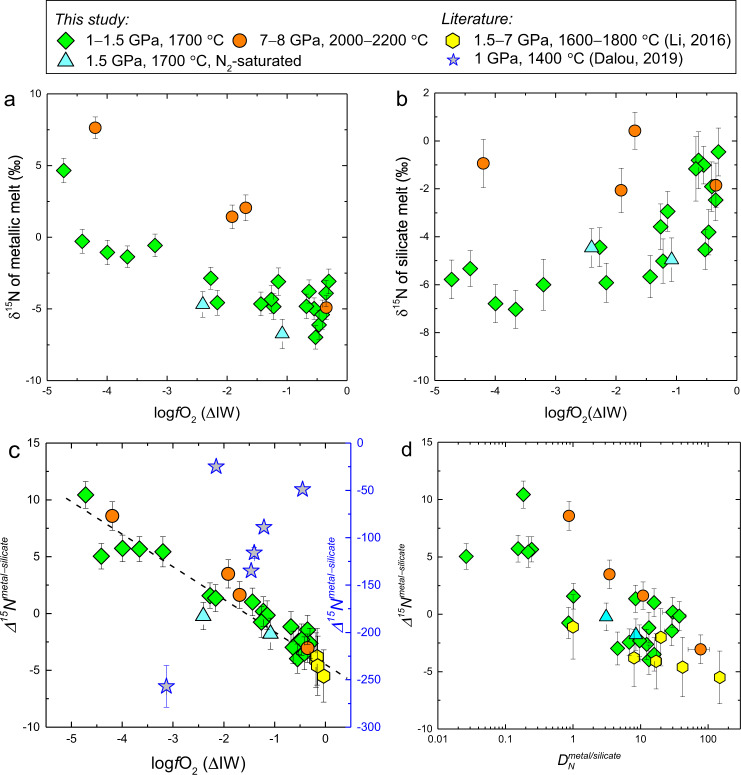
The N-isotopic composition (δ^15^N) of the metallic and silicate melts and metal/silicate N-isotopic fractionation (∆^15^*N*^metal-silicate^). **a** The δ^15^N of metallic melt increase with decreasing *f*O_2_. **b** The δ^15^N of silicate melt generally decrease with decreasing *f*O_2_. **c** The ∆^15^ *N*^metal-silicate^ of this study and Li et al.^35^ are consistent and mainly controlled by *f*O_2_. In the study of Dalou et al.^36^, ∆^15^ *N*^metal-silicate^ vary from −49‰ to −257‰ (the gray-filled blue stars and right-hand blue Y axis). The large N-isotopic fractionations observed in Dalou et al.^36^ could be caused by significant N-loss in their experiments as shown in Supplementary Fig. 5. **d** The ∆^15^ *N*^metal-silicate^ and ${D}_{N}^{{{{{{\rm{metal}}}}}}/{{{{{\rm{silicate}}}}}}}$ obtained in this study and Li et al.^35^ are negatively correlated. More explanations for these data are given in “Methods”. Source data are provided in Supplementary Data 2–4.

The metallic melt δ^15^N (δ^15^N^metal^) increase from −7.0‰ to +7.6‰ with decreasing *f*O_2_, while the silicate melt δ^15^N (δ^15^N^silicate^) decrease from +0.42‰ to −7.0‰ with decreasing *f*O_2_ (Fig. 3a, b). The calculated metal/silicate N-isotopic fractionation factor ∆^15^ *N*^metal-silicate^, which equals δ^15^N^metal^ − δ^15^N^silicate^, increases from −4‰ to +10.4‰ with decreasing *f*O_2_ (Fig. 3c). The *f*O_2_ is the primary factor responsible for the ∆^15^ *N*^metal-silicate^ variation. However, the *f*O_2_ is weakly correlated with the content of Ni, Si, and S in metallic melt, which also shows a weak correlation with the metallic melt δ^15^N (Supplementary Fig. 4). Therefore, light elements and Ni in metallic melt might play a non-negligible role in affecting ∆^15^ *N*^metal-silicate^. Figure 3d shows that ∆^15^ *N*^metal-silicate^ and ${D}_{N}^{{{{{{\rm{metal}}}}}}/{{{{{\rm{silicate}}}}}}}$ are also correlated. The negative correlations between ∆^15^ *N*^metal-silicate^ and *f*O_2_ (Fig. 3c), and between ∆^15^ *N*^metal-silicate^ and ${D}_{N}^{{{{{{\rm{metal}}}}}}/{{{{{\rm{silicate}}}}}}}$ (Fig. 3d), indicate that the ∆^15^ *N*^metal-silicate^ variation can also be explained by the change of N-species in silicate melt as a function of *f*O_2_. The negative ∆^15^ *N*^metal-silicate^ at *f*O_2_ > IW−2 could be caused by the much stronger triple bond of N_2_ in silicate melt than the Fe–N bond in metallic melt, because heavy isotopes tend to be concentrated in species with strong bonds^44^. In contrast, the positive ∆^15^ *N*^metal-silicate^ at *f*O_2_ <IW−2 could reflect a stiffer Fe–N and/or Si–N bond in metallic melt than the N–H or N^3-^–cation bond in silicate melt. Available data^45,46^ show that in gasses and solids at 0–25 °C, the relative bond energy is N≡N (945 kJ/mol) >Si–N (470 kJ/mol) >Fe–N (398 kJ/mol) >N–H (390 kJ/mol), which supports our explanations for the ∆^15^ *N*^metal-silicate^ variation. However, the relative bond energy of Fe–N and Si–N in metallic melt and N–H and N^3-^–cation in silicate melt at the present experimental *P*–*T* conditions cannot be evaluated because of the lack of relevant data.

The present ∆^15^ *N*^metal-silicate^ are consistent with the data of Li et al.^35^ at *f*O_2_ near the IW buffer, but in striking contrast to the results of Dalou et al.^36^, which show that ∆^15^ *N*^metal-silicate^ decreases from −49‰ to −257‰ with *f*O_2_ decreasing from IW–0.5 to IW–3.1 at 1400 °C (Fig. 3c). The large contrast in ∆^15^ *N*^metal-silicate^ between these two studies cannot be explained by the difference in experimental temperature or N-species in silicate melt. We note that in the experiments of Dalou et al.^36^, a large fraction of sample N was lost during the experiment, which may have caused kinetic N-isotopic fractionation, rather than equilibrium fractionation. The negative correlation between ∆^15^ *N*^metal-silicate^ and the N-loss fraction, as observed for the experiments in Dalou et al.^36^ (Supplementary Fig. 5), supports this inference. Kinetic processes can cause significant light element isotopic fractionations even at high temperatures, for example, H-isotopic fractionations with δD variation up to 550‰ having been observed during hydration or dehydration of silicate glasses, melts, and nominally anhydrous minerals at 1000 °C (ref. 47).

The equilibrium mass-dependent isotopic fractionation between two phases is a function of 1/*T*^2^ (ref. 44). We tentatively fit our ∆^15^ *N*^metal-silicate^ along with the data of ref. 35 as a multi-function of *f*O_2_, temperature (K), and all other parameters that potentially affect ∆^15^ *N*^metal-silicate^. We then obtained the following equation to best describe ∆^15^ *N*^metal-silicate^:

2 $${\triangle }^{15}{N}^{{{{{{\rm{metal}}}}}}-{{{{{\rm{silicate}}}}}}}=\frac{-1.6\left(0.1\right)\times {10}^{7}-1.1\left(0.1\right)\cdot \triangle {IW}\times {10}^{7}}{{T}^{2}}({{{{{{\rm{R}}}}}}}^{2}=0.9{{{{{\rm{;}}}}}}{{{{{\rm{\sigma }}}}}}=1.4)$$

In Eq. (2), the light element and Ni content in metallic melt, pressure, and NBO/T are not included as variables, because our trial-and-error fitting yielded that their effects on ∆^15^ *N*^metal-silicate^ are statistically negligible. The negligible effect of pressure on ∆^15^ *N*^metal-silicate^ is consistent with theoretical calculations for carbon, which show that the pressure-dependence of C-isotopic fractionation between Fe-carbide and mantle phases at mantle temperatures is insignificant^48^. Equation (2) allows for calculating ∆^15^ *N*^metal-silicate^ under *T*–*f*O_2_ conditions relevant for planetary core formation, and thus can be used to constrain N-isotopic fractionations between planetary cores and mantles.

### N-partitioning and -isotopic fractionation during Earth’s accretion and core-formation

A multistage core-formation model^49^, which involves the Earth accreting through a series of violent collisions with differentiated impactors, has successfully reproduced Earth’s mantle major and some trace element compositions. The model was further refined by combining it with Grand Tack N-body accretion simulations^15^. In order to investigate N-partitioning and -isotopic fractionation between Earth’s core and mantle, we applied Eqs. (1) and (2) into the multistage model with inputs of the Grand Tack N-body simulation results (Methods). We first tested whether the Earth’s mantle could have obtained its present-day δ^15^N of −5‰ through core-formation alone, if the Earth accreted its 100% mass through the collisions of reduced, EC-like impactors with δ^15^N = −30‰. Supplementary Fig. 7 shows that core-formation alone cannot lead to Earth’s present-day mantle δ^15^N from an initial δ^15^N of −30‰, which contradicts previous arguments based on limited ∆^15^ *N*^metal-silicate^ data^35,36^.

Following the success of the multistage model with inputs of the Grand Tack N-body simulation results, we modeled the N-behavior by considering that the Earth accreted its first 60% mass through the collisions of reduced, EC-like impactors, and its last 40% mass through the collisions of increasingly oxidized impactors. Reduced, EC-like impactors formed at heliocentric distances of <0.9–1.2 AU with δ^15^N = −30‰, while increasingly oxidized impactors originated from great heliocentric distances (1.2–3 AU). Since the solar system δ^15^N increases with the heliocentric distance^4,20^, the increasingly oxidized impactor δ^15^N should increase from −30‰ (EC’s value) to a slightly positive value at 3 AU. We used a δ^15^N value of +5‰ for the last impactor, which added Earth’s last 10% mass through the Moon-forming giant impact, and a δ^15^N of +5‰ represents the mixing of ECs (δ^15^N = −30‰) and CCs (δ^15^N = +40‰) with a same N content and mass ratio of ~1:1 as constrained by Mo isotopes for the Moon-forming giant impactor^19^. In the N-body simulations, after Earth accreted its 60% mass, a small mass of completely oxidized or CI chondrite-like materials, which contains ~1000 ppm N with δ^15^N = +40‰ and formed from beyond 6–7 AU, was delivered to Earth’s magma ocean^50^. No metal–silicate segregation occurred when CI chondrite-like materials added in the magma ocean, but the N in the silicate magma ocean participated in the subsequent core-formation events when metal-bearing impactors were accreted^15^. Large and oxidized impactors may hold more N than small and reduced ones, and the N is mainly stored in the cores^42^. In our model, we varied the bulk N-content from 50 ppm for small and reduced impactors to 150 ppm for large and oxidized impactors. We also considered the other factors that potentially affect the N-content and -isotopic composition of Earth’s different reservoirs, which include the formation of a proto-atmosphere, the equilibrium degree between the silicate magma ocean and the proto-atmosphere, catastrophic loss of the proto-atmosphere during each collisional accretion, and the light element composition of Earth’s core. Details for the setup of our model are given in “Methods”.

**Fig. 4:**
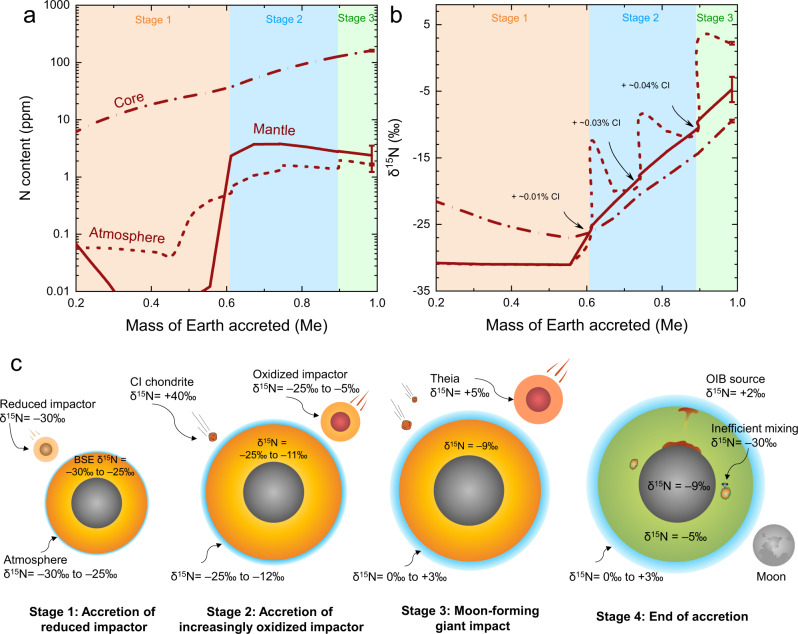
Earth’s heterogeneous accretion of nitrogen during its main accretion phase. **a**, **b** The N-content and -isotopic composition of the proto-Earth’s core, mantle, and atmosphere as a function of mass fraction accreted. At the end of Earth’s accretion, the N-content and δ^15^N of the proto-Earth’s mantle are ~2.4 ppm and −4.7‰, respectively, consistent with previous estimates for the present-day mantle^5,6,8^. The N-content of the proto-Earth’s atmosphere is ~1.7 ppm, consistent with previous estimates for Earth’s early atmosphere^52^ (note that the ppm N in the atmosphere is based on the atmosphere N-mass normalized to the silicate Earth mass). The delivery of oxidized, CI chondrite-like materials plays an important role in enhancing the proto-atmosphere δ^15^N from −30‰ to 0‰–+3‰, which are close to Earth’s surface δ^15^N (atmosphere + crust; Fig. 1). Earth’s core contains more than 90% of Earth’s bulk N. The error bars at ~99% accretion were based on ±2σ for ${D}_{{{{{\rm{N}}}}}}^{{{{{{\rm{metal}}}}}}/{{{{{\rm{silicate}}}}}}}$ and ∆^15^ N^metal-silicate^. **c** Illustration showing the delivery of N to the proto-Earth by first reduced, EC-like impactors and then increasingly oxidized impactors. The Earth accreted its first 60% mass from EC-like planetesimals/embryos with δ^15^N of −30‰ (Stage-1). After acquiring its 60% mass, the Earth started to accrete from increasingly oxidized impactors which have δ^15^N varying from −30‰ to +5‰, and from minimal CI chondrite-like materials which have an average δ^15^N of +40‰ (Stages-2 and −3). As shown in Stage-4, inefficient mantle mixing of EC-like impactor materials may explain the negative δ^15^N of some deep mantle diamonds^25^, while a long-term preservation of oxidized impactor materials at Earth’s core–mantle boundary may explain the positive δ^15^N of OIB^30^.

The model results are shown as a function of accretion mass fraction in Fig. 4a, b. At the end of Earth’s accretion, the N-content and δ^15^N of the proto-Earth’s mantle are ~2.4 ppm and about −4.7‰, respectively, both of which are consistent with the present-day mantle estimates of ~1–3 ppm^5,6,8,51^ and −5 ± 2‰ (ref. 22). The N-content of the proto-atmosphere is ~1.7 ppm (normalized to Earth’s mantle mass), i.e., ~1.7 times present-day atmospheric N-mass, which well matches the N-mass estimated for Earth’s early atmosphere^52^. In addition, the proto-atmosphere δ^15^N are 0‰ to +3‰, which are consistent with Earth’s surface reservoir δ^15^N (crust + atmosphere; Fig. 1). Although the totally added CI chondrite-like materials were only ~0.1% of Earth’s mass, they play a significant role in enhancing the proto-atmosphere δ^15^N to the values of 0‰ to +3‰ (Fig. 4b). This is because most N in the shallow magma ocean, sourced from CI chondrite-like materials, were released into the proto-atmosphere due to the rather low N-solubility in silicate melt under relatively oxidized conditions^53^.

We noticed that the largely negative δ^15^N features of some deep mantle diamonds (Fig. 1) could be primordial, representing the relicts of inhomogeneous mantle mixing of EC-like impactors, as suggested previously^25^. The positive δ^15^N features of OIB mantle source could also be primordial, representing a long-term preservation of materials from oxidized impactors, such as those from the Moon-forming giant impactor (Fig. 4c). Accordingly, our model can explain the observed N-content and δ^15^N of Earth’s different reservoirs (Fig. 4c). It is also worth noting that Earth’s core may contain ~160 ppm N (Fig. 4a), which accounts for more than 90% of Earth’s bulk N, and that Earth’s core may have δ^15^N close to −9‰ (Fig. 4b), more negative than those of Earth’s silicate reservoirs.

Our model demonstrates that the N-content and δ^15^N of Earth’s different reservoirs were determined by complex processes but happening naturally during Earth’s main accretion phase. We recognized that during Earth’s multistage accretion, the processes such as collision-induced catastrophic atmosphere loss and the degree of silicate magma ocean–atmosphere equilibrium remain loosely constrained; however, the target N-content and δ^15^N of Earth’s different reservoirs can still be obtained if the used parameters in our model were varied simultaneously (Supplementary Figs. 8–14 and “Methods”). We thus suggest that Earth established its N-inventories and -isotopic signatures through heterogeneous accretions of impactors formed at different heliocentric distances in the solar system.

### Implications for the distribution and origin of Earth’s major volatiles

**Fig. 5:**
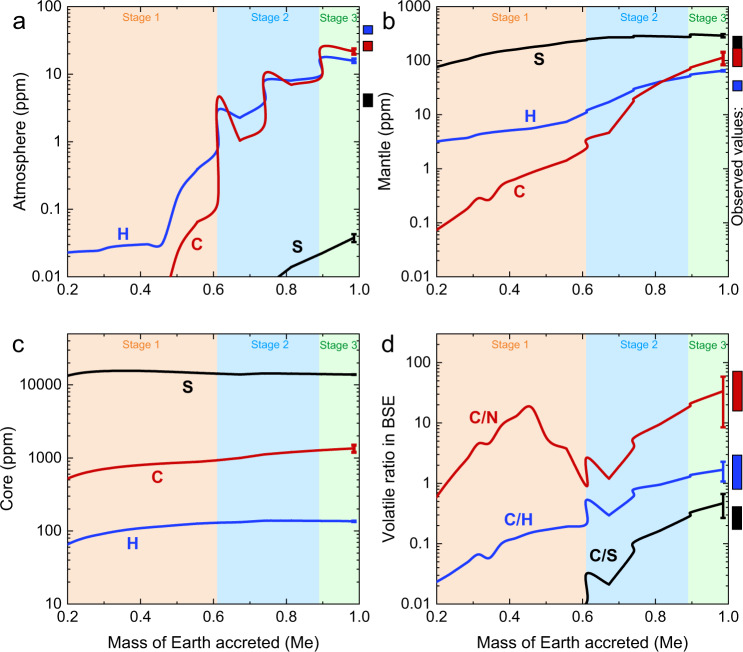
Earth’s heterogeneous accretion of C–H–S during its main accretion phase. **a**–**c** The C–H–S contents in proto-Earth’s atmosphere, mantle, and core as a function of mass fraction accreted. Note that the C–H–S contents in the proto-atmosphere is based on the proto-atmospheric C–H–S-mass normalized to the silicate Earth mass. **d** The C/N, C/H, and C/S ratios in the BSE (atmosphere + mantle) change as the mass of Earth accreted. The observed C and H contents in Earth’s present-day reservoirs were taken from ref. 51, and the S content in Earth’s present-day atmosphere and mantle were taken from ref. 59 and ref. 58, respectively. The error bars at ~99% accretion were based on ±2σ for the used metal/silicate partition coefficients. Note that the atmosphere in panel (**a**) represents Earth’s surface reservoir including atmosphere and crust, due to the condensation of water and deposit of CO_2_ from atmosphere to crust. Source data are provided in Supplementary Data 5.

**Fig. 6:**
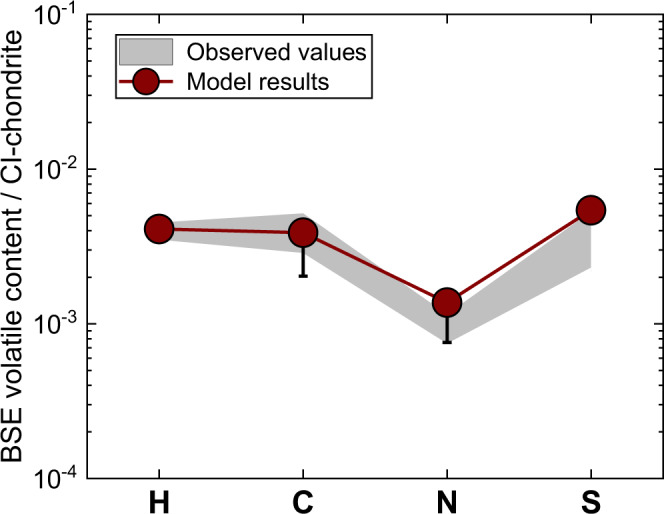
Comparison between our modeled volatile contents and the observed values in the bulk silicate Earth (BSE). The observed C, N, and H contents in Earth’s present-day BSE were taken from ref. 51, and the S content in BSE were calculated based on refs. 58,59. Note that our modeled H–C–N–S contents are in good agreement with the observed values in the BSE, indicating that the Earth acquired its major volatile inventory through its heterogeneous accretion. Source data are provided in Supplementary Data 5.

Figure 4 illustrates that Earth first acquired its N from EC-like impactors, and then acquired additional N from increasingly oxidized impactors and minimal CI chondrite-like materials before and during the Moon-forming giant impact. Similarly, the Earth could have also accreted its other major volatiles from these objects. Both ECs and CI chondrites contain significant amounts of H, C, and S^4,54,55^; therefore, H, C, and S may have also been delivered and participated in Earth’s core-formation during Earth’s main accretion phase. We applied our above “N-accretion model” (Fig. 4) to C–H–S to constrain the distribution and origin of C–H–S in Earth’s different reservoirs (“Methods” and Supplementary Data 5). Figure 5 shows that at the end of Earth’s accretion, the proto-atmosphere contains ~16 ppm H, ~22 ppm C, and negligible S; the proto-mantle contains ~65 ppm H, ~112 ppm C, and ~290 ppm S. After Earth’s accretion, the H_2_O in the proto-atmosphere would condensate to forming primitive oceans^56^ and the CO_2_ in the proto-atmosphere would deposit as carbonates in Earth’s surface (atmosphere +  crust)^57^. Our model yields C-contents in the proto-atmosphere and proto-mantle consistent with the observed values in Earth’s present-day surface and mantle^51^. Nevertheless, our model yields lower H- and S-contents in the proto-atmosphere but higher H- and S-contents in the proto-mantle (Fig. 5a, b), compared to the estimates for Earth’s present-day surface and mantle^51,58,59^. However, the obtained C–S–H–N contents and C/H, C/S, and C/N ratios in the BSE are in good agreement with the estimates for the present-day BSE (Figs. 5d, 6). This implies a contribution of Earth’s mantle to surface in H and S through degassing after Earth’s accretion. In addition, our model results show that Earth’s core is a major reservoir for C–H–S (Fig. 5c), as in the case for N (Fig. 4a), which includes ~1300 ppm C, ~160 ppm H, and ~1.4 wt.% S, consistent with the estimates for Earth’s core^60^.

We performed mass-balance calculations to constrain the contributions of different types of impactors to Earth’s major volatile budget. Our results (Supplementary Data 6) show that the firstly accreted EC-like impactors supply ~45%, ~72%, ~38%, and ~12% of Earth’s H, S, C, and N, respectively; the lately accreted increasingly oxidized impactors supply ~46%, ~28%, ~60%, and ~87%, respectively; whereas, the oxidized CI chondrite-like materials supply less than 10% of Earth’s major volatiles. The fraction of H (~45%) supplied by EC-like impactors in our model is lower than that (~70%) in a previous model^54^, which used a simple EC–CC mixing to explain the H-isotopic composition in the BSE. However, the disparity could be resolved by the lately accreted increasingly oxidized impactors, because they are composed of mixed EC and CC, as suggested by Mo isotopes^19,61^.

Conclusively, our results (Figs. 4–6) demonstrate that the Earth acquired its major volatiles from different cosmochemical reservoirs during its main accretion phase, rather than from a single giant impactor or at its late accretion stages. Combined contributions of reduced and oxidized impactors and minimal CI chondrite-like materials, formed at different heliocentric distances in the solar system, to Earth’s major volatile inventory agree with the observed geochemical and cosmochemical constraints^5,19,21,32,54^ and dynamic models of planetary formation^2,15^.

## Methods

### Starting materials

Starting materials included synthetic silicates and mixtures of metal Fe + Fe_7_N_3 _± FeS ± Si powder. The first three silicates (refer to as G-1, G-2, and G-3) had major element compositions similar to those of mid-ocean ridge basalts (MORB), but the FeO in G-3 silicate was replaced by MgO and CaO (i.e., FeO-free). A fourth silicate (G-4) also had a basaltic composition but a large fraction of its MgO was replaced by FeO. A fifth silicate (G-5) had major element compositions similar to those of Earth’s mantle pyrolite. All silicates were prepared from analytical grade oxides and carbonates. To minimize absorbed water, SiO_2_, TiO_2_, Al_2_O_3_, and MgO powder were each fired over night at 1000 °C, MnO_2_ at 400 °C, CaCO_3_ at 200 °C, and Na_2_CO_3_ and K_2_CO_3_ at 110 °C. After drying, all these oxides and carbonates were first mixed and ground in ethanol in an agate mortar and dried at room temperature overnight. Well mixed powders were decarbonated at 1000 °C. Then the FeO powder, stored in a vacuumed oven at 100 °C, was added to the mixture and further ground in ethanol in an agate mortar, and then dried at room temperature overnight. G-1 to G-3 silicates were synthesized as glasses at 1600 °C and powdered before use. In addition, G-1 and G-2 silicate powders were reduced at 1000 °C and *f*O_2_ of FMQ–2 for 24 h using a CO–CO_2_ gas mixing furnace. G-4 and G-5 silicates were not synthesized as glass. The major element compositions of G-1 to G-5 silicates are provided in Supplementary Data 1. The Fe+Fe_7_N_3_±FeS±Si powder was prepared from high-purity Fe, Fe_7_N_3_, Si, and FeS powder, homogenized by grinding under ethanol in an agate mortar, and dried at room temperature. Nominally, Fe_7_N_3_ was the only N source in the sample. The bulk sample N-contents were prepared to be at 1000–2000 ppm, far below the N-solubility in Fe-rich metallic melt under *P*–*T* conditions adopted in this study^37^. For comparison, in two experiments LY24 and LY25, ~50 wt.% silicate and ~50 wt.% Fe_7_N_3_ were used to produce a large amount of N_2_ gas in the graphite capsules. In addition, different amounts of Mg(OH)_2_ were added in a few experiments to obtain hydrous conditions. All of the dried starting materials were stored in a vacuumed oven at 100 °C for >24 h before loading into graphite capsules, zirconia-lined Pt_95_Rh_05_ capsules, or graphite-lined Pt_95_Rh_05_ capsules for high-pressure experiments.

### High-pressure experiments

All experiments at 1–1.5 GPa and 1700 °C were conducted in an end-loaded, solid media piston cylinder apparatus, using 0.5-inch diameter talc–Pyrex assemblies with stepped graphite heaters (Supplementary Data 2). For these experiments, starting materials of 30–50 wt.% metallic mixture and 50–70 wt.% silicate powder were loaded into zirconia-lined or graphite-lined Pt_95_Rh_05_ capsules. The use of zirconia-lined Pt_95_Rh_05_ capsules was to avoid the presence of C in the sample. The hot piston-in method was used to pressurize the assembly. Pressure was calibrated against the quartz–coesite and kyanite–sillimanite transitions, and a friction correction of 18% was applied to the nominal pressure. The pressure uncertainty is estimated to be better than 5% relative. We used W_97_Re_03_–W_75_Re_25_ thermocouples and temperature was controlled to ±2 °C and is accurate to ±10 °C. The experimental durations ranged from 1 to 3 h. All experiments were quenched to below 100 °C within 10–20 s by turning off electric power to the graphite heaters.

The experiments at 2000–2200 °C and 5–8 GPa were conducted in a 1000-ton DIA-type multi-anvil apparatus. The starting material was loaded into graphite capsules. Tungsten carbide (WC) anvils with 8-mm truncated edge lengths, together with a Cr_2_O_3_-doped MgO octahedron (14 mm edge length) as the pressure medium, were used to generate high pressures. The furnace is composed of a stepped cylindrical graphite or LaCrO_3_ heater and a ZrO_2_ thermal insulator. Sample pressures were estimated from the hydraulic pressure using calibrations based on the phase transitions of Bi, ZnS, and Mg_2_SiO_4_ polymorphs. Temperatures were measured using a W_95_Re_05_–W_74_Re_26_ thermocouple whose junction was located near the end of the graphite capsule. The cell assemblies were first pressurized to target pressures at room temperatures in 4 h and then heated to target temperatures at a rate of 100 °C/min. The temperature fluctuation during the experiments was approximately ±10 °C, and the experimental durations ranged from 20 to 40 mins. All experiments were quenched by switching off the electric power, and then decompressed to ambient pressure for more than 16 h.

All recovered sample capsules were sectioned longitudinally into two halves. One half was mounted in epoxy and polished for electron microprobe and Raman spectroscopy analysis, and the other half prepared for noble gas mass spectrometry analysis.

### Electron microprobe analysis

Major element compositions of the quenched metallic and silicate melts were measured with a JEOL JXA–8200 microprobe. The analyses were performed in wavelength-dispersive mode, and a PAP matrix correction was applied to the raw data. The metallic melts were analyzed with 20 kV acceleration voltage and 20 nA beam current, whereas the silicate melts were analyzed with 15 kV/10 nA. Natural and synthetic standards were used to calibrate the instrument. For the metallic melt analysis, Fe, Si, Ni, and Cu were calibrated on pure metals, S was calibrated on a synthetic pyrrhotite with well-known Fe: S ratio, and O was calibrated on magnetite. For the analysis of C in metallic melt, the samples and standards were uncoated but surrounded with silver-bearing conductive varnish to avoid charging, and Fe_3_C was the standard. For the silicate melt analysis, Na was calibrated on albite, Ca on wollastonite, K on orthoclase, Ti and Mn on ilmenite, Si on enstatite, Mg on forsterite, Al on spinel, P on GaP, and Fe on metallic Fe. Sulfur in the quenched silicate melts was analyzed with 50 nA beam current and 60 s peak counting time using a pyrrhotite standard. A defocused beam of 10 or 20 µm diameter was used for all standardizations and sample measurements.

### Noble gas mass spectrometry analysis

The coexisting metallic melt and silicate melt in one half of all samples were physically separated and measured for N-content and -isotopic composition, using a modified noble gas mass spectrometry at the Atmosphere and Ocean Research Institute, the University of Tokyo, which has a capacity to precisely measure N-content and -isotopes at the sub-Nanomole levels^62^. Big metal blobs and clean silicates were separated readily under binocular microscope, except for run PYH01 synthesized at 5 GPa and 2200 °C (Supplementary Data 2), which only contained small metal grains (10–30 µm) being heterogeneously distributed in the quenched silicate melt. For this sample, we did not measure its N-content and -isotopic composition.

Silicate chips with a mass of ~1 mg and metal chips with a mass of ~0.1 mg were prepared for each sample. Each silicate or metal was loaded into a small quartz glass tube, which was then loaded into a large quartz glass container. The large quartz glass sample container was then placed in a furnace equipped with a tungsten wire. The sample container was baked out at 150 °C overnight under vacuum to remove any atmospheric N. A high-vacuum line was used for N gas extraction and purification. The vacuum line is basically composed of four parts: a gas-extraction part connected to the sample container, a gas-purification line (AQ-line) including cold trap-1, cold trap-2, a copper oxide finger (CuO) and a Pt finger, a vacuum line (AC-line), and a vacuum line directly connected to the mass spectrometer VG3600 (CE-line) for N-measurements. The AQ vacuum line is connected to a quadrupole mass spectrometer for checking whether N-intensity is appropriate for measurements. The vacuum lines are evacuated by a turbo molecular pump and an ionic pump. About 0.2 torr and 1 torr O_2_ was produced for silicate sample and metal sample, respectively, by heating a copper oxide finger (CuO) to 850–900 °C. The produced O_2_ was sealed in the quartz glass sample container and used for oxidizing the sample. All samples were firstly heated to 200 °C to remove any potential surficial contamination and to check the leakage of the system. During heating, the excess O_2_ in the AQ line was absorbed by the Cu finger through decreasing temperature of the finger to 600 °C. After the excess O_2_ was absorbed, the AQ line was evacuated, and the copper oxide finger (CuO) was reheated to 850 °C to produce O_2_. After oxidizing the samples at 200 °C for 30 min, the gases in the system produced at 200 °C were then directed to the purification vacuum line (AQ) by opening the valve of the sample container for 2 min. Condensable gases such as carbon dioxide and water were trapped using liquid N at cold trap-1. The non-condensable gases such as carbon monoxide, hydrocarbons, and hydrogen in the AQ line were then reacted for 5 min with pure O_2_, catalyzed by a platinum foil at 1000 °C. Carbon monoxide, hydrocarbons, and hydrogen were oxidized to CO_2_ and H_2_O by the pure O_2_. After reaction for 5 min, excess O_2_ was again resorbed by copper by decreasing the furnace temperature to 600 °C for 20  min and eventually to 450 °C for another 20 min. During this time, the produced gases such as CO_2_ and H_2_O were trapped using liquid N at cold trap-2. The pressure in the purification line, which is measured by a capacitance manometer, is no more than 0.0001 torr higher than the baseline pressure. A quadrupole mass spectrometer (QMS; HAL201, Hiden Analytical) was used to check whether the sample volume is proper for analysis through determining the sample size. If the N_2_ intensity in QMS is in the appropriate range, then the purified gases would be measured using the high-sensitivity static vacuum mass spectrometer (a modified VG3600, VG Micromass Ltd.). The determined concentration of N released at 200 °C was negligible (less than 1 ppm). After the measurements of gases released at 200 °C, following the same procedure, the samples were then heated to 1200 °C and kept for 30 min for glasses and 60 min for metals to ensure that the samples were completely oxidized for releasing N. The purification and measurement procedure for gases released at 1200 °C was the same as the procedure at 200 °C. After each measurement finished, the VG3600 mass spectrometer was evacuated for at least 20 min.

The running standard gas for N-isotopes was reserved in a large metal container attached to CE-line. The N-isotopic ratio of the standard gas was measured before and after the sample analysis, and the N-isotopic ratio of the standard gas was checked periodically by comparison with that of the local air. After the standard/sample gas was introduced into the VG3600 mass spectrometer, the intensities of 28 and 29 were measured for 15 times, and the 28/29 ratio was extrapolated by the mass spectrometer, with a system error of ~0.3‰. Repeated analysis of the standard in a day showed that the overall reproducibility was better than 0.5‰ for N-isotopes, and repeated analysis of a same sample yielded consistent results with uncertainties of <5% relative. The N-isotopic ratio of the air was analyzed for a number of times during the course of analysis, which gave an average δ^15^N value of −0.03‰. The concentration and isotopic composition of N in each sample, released at 1200 °C, was corrected by subtracting the hot blank (i.e., background), and the hot blank was analyzed with glass tube that contained no sample but using the same heating duration, same amounts of oxygen, and same purification procedure as in the case for measuring the samples. An average N-isotopic composition of +2.99‰ ± 0.70‰ is obtained for the hot blank.

The error (σ) of the measured N-concentration was calculated based on the error of the N-concentration measured by the VG3600 mass spectrometer, the error of the hot blank, and the error of weighing the sample mass, which can be defined as:

3 $${\sigma }_{{{{{\rm{N}}}}}}({{{{{\rm{ppm}}}}}})={{{{{\rm{N}}}}}}({{{{{\rm{ppm}}}}}})\times \sqrt{{\left(\frac{{\sigma }_{{{{{\rm{N}}}}}2}({{{{{\rm{mole}}}}}})}{{{{{{\rm{N}}}}}}_{2\,{{{{{\rm{original}}}}}}}\left({{{{{\rm{mol}}}}}}\right)-{{{{{\rm{N}}}}}}_{2\,{{{{{\rm{blank}}}}}}}({{{{{\rm{mol}}}}}})}\right)}^{2}+{\sigma }_{{{{{{\rm{weighing}}}}}}}^{2}}$$

In Eq. (3), N (ppm) is the blank-corrected N-concentration in the sample; and the error of sample weighing (*σ*_weighing_) is usually 1% relative; N_2original_ (mol) is the uncorrected N-concentration measured by the VG3600 mass spectrometer; *σ*_N2_ (mole) is the error of background-corrected N-concentration in the sample, which can be defined as:

4 $${\sigma }_{{{{{{\rm{N}}}}}}_{2}}({{{{{\rm{mol}}}}}})=\sqrt{{\sigma }_{{{{{{\rm{N}}}}}}_{2}{{{{{\rm{original}}}}}}}^{2}+{\sigma }_{{{{{{\rm{N}}}}}}_{2}{{{{{\rm{blank}}}}}}}^{2}}$$

5 $${\sigma }_{{{{{{\rm{N}}}}}}_{2}{{{{{\rm{blank}}}}}}}({{{{{\rm{mole}}}}}})={{{{{\rm{N}}}}}}_{2\,{{{{{\rm{blank}}}}}}}\left({{{{{\rm{mol}}}}}}\right)\times {\sigma }_{{{{{{\rm{N}}}}}\; {{{{{\rm{sensitivity}}}}}}\; {{{{{\rm{in}}}}}}\; {{{{{\rm{air}}}}}}}}$$

where the ${\sigma }_{{{{{{\rm{N}}}}}\; {{{{{\rm{sensitivity}}}}}}\; {{{{{\rm{in}}}}}}\; {{{{{\rm{air}}}}}}}}$ is about 9.9%. The error of N-isotopic composition of the sample was calculated based on the error of sample gas δ^15^N (‰) measured by the VG3600 mass spectrometer, the error of reproducibility of standard N_2_ (0.5‰), and error of δ^15^N of the air (0.43‰), which can be defined as:

6 $${\sigma }_{{\delta }^{15}N({{\textperthousand }})}=\sqrt{{\sigma }_{{{{{{{\rm{\delta }}}}}}}^{15}{{{{{\rm{N}}}}}}{\left({{\textperthousand }}\right)}_{{{{{{\rm{MS}}}}}}}}^{2}+{\sigma }_{{{{{{{\rm{\delta }}}}}}}^{15}{{{{{\rm{N}}}}}}{\left({{\textperthousand }}\right)}_{{{{{{\rm{Standard}}}}}}}}^{2}+{\sigma }_{{{{{{{\rm{\delta }}}}}}}^{15}{{{{{\rm{N}}}}}}{\left({{\textperthousand }}\right)}_{{{{{{\rm{Air}}}}}}}}^{2}}$$

7 $${\sigma }_{{{{{{{\rm{\delta }}}}}}}^{15}{{{{{\rm{N}}}}}}{\left({{\textperthousand }}\right)}_{{{{{{\rm{MS}}}}}}}}=\sqrt{{\sigma }_{{28/29{{{{{\rm{N}}}}}}}_{{{{{{\rm{Standard}}}}}}-1}}^{2}/2+{\sigma }_{{28/29{{{{{\rm{N}}}}}}}_{{{{{{\rm{MS}}}}}}}}^{2}+{\sigma }_{{28/29{{{{{\rm{N}}}}}}}_{{{{{{\rm{Standard}}}}}}-2}}^{2}}/2$$

where ${\sigma }_{{{{{{{\rm{\delta }}}}}}}^{15}{{{{{\rm{N}}}}}}{\left({{\textperthousand }}\right)}_{{{{{{\rm{MS}}}}}}}}$ is the error of δ^15^N (‰) of sample gas measured by the VG3600 mass spectrometer; ${\sigma }_{{{{{{{\rm{\delta }}}}}}}^{15}{{{{{\rm{N}}}}}}{\left({{\textperthousand }}\right)}_{{{{{{\rm{Standard}}}}}}}}$ is the error of the reproducibility of the standard gas; repeated analysis of the standard in a day showed that the overall reproducibility was better than 0.5‰; ${\sigma }_{{{{{{{\rm{\delta }}}}}}}^{15}{{{{{\rm{N}}}}}}{\left({{\textperthousand }}\right)}_{{{{{{\rm{Air}}}}}}}}$ is the error of the δ^15^N (‰) value of the air, which is 0.43‰; ${\sigma }_{{28/29{{{{{\rm{N}}}}}}}_{{{{{{\rm{MS}}}}}}}}$ is the standard deviation of 15 times measurements of 28/29 ratio of sample gas extrapolated by the mass spectrometer, which is approximately 0.3‰; ${\sigma }_{{28/29{{{{{\rm{N}}}}}}}_{{{{{{\rm{Standard}}}}}}-1}}$ and ${\sigma }_{{28/29{{{{{\rm{N}}}}}}}_{{{{{{\rm{Standard}}}}}}-2}}$ are the standard deviation of 15 times measurements of 28/29 ratio of the standard gas before and after each sample analysis.

### Raman spectroscopy analysis

To obtain information on the speciation of N–C–H–O in the silicate melt, Raman spectra of some silicate glasses were collected. Micro-Raman spectra were recorded in back-scattering geometry using a Horiba LabRAM HR UV spectrometer with CCD detector, 1800 mm^−1^ grating, 50× objective, and confocal mode. A confocal pinhole of 100 μm was used, which limits spectral resolution to 3.5 cm^−1^. The 514.5 nm line of an Ar+ ion laser with an output power 0.1 W was used for excitation. Spectra were collected from 200 to 4500 cm^−1^ with an acquisition time of 2 × 300 s for each range, in order to detect N–C–H–O species with very low concentrations. Typical Raman spectra of some silicate glasses at *f*O_2_ of ~IW−0.5 are shown in Supplementary Fig. 2. We encountered significant fluorescence when measuring the reduced silicate melts. Such fluorescence could significantly mask the weak peaks of N–C–H–O species; we therefore only reported the Raman spectra that were not affected by fluorescence (Supplementary Fig. 2). Actually, the variation of N–C–H–O species in silicate melt as a function of *f*O_2_ has been extensively measured and discussed in the literature^36,38,39,53,63,64^, and their results are consistent with one another. Here we only cited these previous studies to discuss the effect of the variation of N-species in silicate melt on the N-isotopic fractionation (see the main text).

### Major element compositions of experimental products and calculation of sample *f*O_2_

In all experiments, the basaltic melts were quenched into glasses, while the mantle pyrolitic melts had a dendritic texture (Supplementary Fig. 1). Major element compositions of the quenched metallic and silicates are given in Supplementary Data 3, 4. The ratio of non-bridging oxygens to tetrahedral cations (NBO/T) of the quenched silicate melt was 0.2–3.1, and the Fe-rich metallic melts contained 87.0–98.8 wt.% Fe, 0–6.8 wt.% Si, 0–1.8 wt.% S, and 0–11.5 wt.% C.

The *f*O_2_ prevailing in our experiments was calculated from the coexistence of Fe-rich metallic melt and silicate melt with finite FeO content using the following equilibrium:

8 $${{{{{\rm{FeO}}}}}}({{{{{\rm{silicate\; melt}}}}}})={{{{{\rm{Fe}}}}}}({{{{{\rm{metallic\; melt}}}}}})+{1/2{{{{{\rm{O}}}}}}}_{2}$$

from which the *f*O_2_ relative to the Fe-FeO buffer at any given *P*–*T* can be defined as:

9 $$\Delta {{{{{\rm{IW}}}}}}=2{{\log }}({a}_{{{{{{\rm{FeO}}}}}}}/{a}_{{{{{{\rm{Fe}}}}}}})=2{{\log }}({X}_{{{{{{\rm{FeO}}}}}}}{{{{{{\rm{\gamma }}}}}}}_{{{{{{\rm{FeO}}}}}}}/{X}_{{{{{{\rm{Fe}}}}}}}{{{{{{\rm{\gamma }}}}}}}_{{{{{{\rm{Fe}}}}}}})$$

*a*_FeO_ represents the activity of FeO in the silicate melt; *a*_Fe_ represents the activity of Fe in the metallic melt; *X*_FeO_ and *X*_Fe_ are molar fractions of FeO in the silicate melt and Fe in the metallic melt, respectively; γ_FeO_ and γ_Fe_ are activity coefficients of FeO in the silicate melt and Fe in the metallic melt, respectively. Calculations of *f*O_2_ using both ideal (γ_FeO_  = 1 and γ_Fe_ = 1; ideal *f*O_2_) and non-ideal solution models (non-ideal *f*O_2_) were performed. The *f*O_2_ calculation using the non-ideal solution model was performed assuming γ_FeO_ = 1.5 (refs. 17,65). γ_Fe_ was calculated using the ε-approach and the online “Metal Activity Calculator” (http://www.earth.ox.ac.uk/~expet/metalact/) provided by the University of Oxford, which take into account the non-ideal interaction between all components in the Fe-rich metallic melt^66^. The calculated non-ideal *f*O_2_ values are between IW and IW–5. The calculated ideal *f*O_2_ values are between IW–0.7 and IW–6, which are 0.7–1 log units lower than the non-ideal *f*O_2_ values. The calculated *f*O_2_ values decrease with decreasing the FeO content of the silicate melt or increasing the Si content of the metallic melt, consistent with previous results^17^.

### Sample N-content and δ^15^N

The δ^15^N of the starting Fe_7_N_3_ was −7.9 ± 1‰. The N-contents of the metallic and silicate melts are 43–14293 ppm and 44–4620 ppm (by wt.), respectively (Supplementary Data 3, 4). In two experiments (LY24 and LY25; Supplementary Data 2), where ~50 wt.% Fe_7_N_3_ was added in the starting material, the metallic melts contained ~1.1 and 1.4 wt.% N, respectively. These values are comparable to the N-solubility in Fe-rich metallic melts determined under similar conditions^37^, implying that these two experiments must have been N_2_-saturated. The δ^15^N of metallic melts ranged from −7.0‰ to +7.6‰, and the δ^15^N of silicate melts ranged from −7‰ to +0.42‰ (Supplementary Data 3, 4). Except for the two N_2_-saturated experiments, mass-balance calculations of bulk sample N content and δ^15^N were performed for all other experiments (Supplementary Data 2). The results show that the bulk sample N-contents vary from 650 to 3000 ppm, with most values (780–2200 ppm) generally consistent with the N-mass added in the starting materials. Most of the bulk sample δ^15^N values vary between −3‰ and −7‰, with the δ^15^N values of three multi-anvil experiments above +0.2‰ (N-18, N-19, and N-20; Supplementary Data 2). The deviation of the bulk sample δ^15^N from that of the starting Fe_7_N_3_ could be explained by air N_2_ contamination when loading silicate powder into the graphite capsule, the presence of N in the starting metallic Fe, and/or δ^15^N inhomogeneity of the starting Fe_7_N_3_. Nevertheless, the positive δ^15^N values of the three multi-anvil experiments are difficult to explain. However, as shown in the main text, the calculated ${D}_{N}^{{{{{{\rm{metal}}}}}}/{{{{{\rm{silicate}}}}}}}$ and ${\triangle }^{15}{{{{{\rm{N}}}}}}^{{{{{{\rm{metal}}}}}}-{{{{{\rm{silicate}}}}}}}$ of these three experiments are fully consistent with those of the other experiments.

### N-loss during experiments?

Previous studies showed that N-loss occurred during the run in some of the high *P*–*T* experiments^36,37,40,41^. The N-loss could be either related to the storage of some N in the porosity of the graphite capsules or to N diffusive loss through the graphite (-Pt) capsule walls^41^. We note that in previous experiments, ~0.5–2 wt.% N was put in the starting materials, so that the samples were usually saturated with a N_2_ vapor phase. However, in our experiments, we added only ~1000–2000 ppm N in the starting materials, which are far below the solubility limit^37^. Our experiments were therefore not saturated with a N_2_ vapor phase, except for two experiments (LY24 and LY25; Supplementary Data 2). As mentioned above, the reconstructed bulk N content in our samples ranged from 650 to 3000 ppm, with most values between 780 and 2200 ppm and consistent with the N-mass added in the starting materials (Supplementary Data 2). We therefore believe that N-loss in our experiments was limited. Some experiments, as shown in Supplementary Data 2, even gained N, which could be caused by air N_2_ contamination during loading sample materials into the capsules, and the presence of N in the starting metallic iron. And our experiments LY24 and LY25 were still saturated with a N_2_ vapor after running 90 mins. The factors that control the N-loss or the N-loss rates are elusive, but at least, the absence of a N_2_ vapor phase should effectively suppress the N-loss rates or the storage of N_2_ gas in the porosity of the graphite capsule walls.

### Attainment of equilibrium partitioning

The experimental durations ranged from 60 to 180 mins at 1700 °C, and from 10 to 20 mins at 2000–2200 °C. The observation that ∆^15^ *N*^metal-silicate^ are time-independent over a small *f*O_2_ range (Supplementary Fig. 6) demonstrates 60 mins being sufficient for approaching metal/silicate N-isotopic equilibrium at 1700 °C. A time series of experiments are usually not performed at temperatures of 2000–2200 °C, because the diffusion of elements at such high temperatures are thought to be very fast. If metal/silicate N-isotopic equilibrium at 1700 °C was approached within 60–180 min, there is no doubt that N-isotopic equilibrium at 2000–2200 °C must have also been approached within 10–20 min, because the diffusion coefficient of an element in a given system increases by orders of magnitude with temperature increasing from 1700 to 2000–2200 °C. In addition, as shown in Fig. 2 and summarized in Eq. (1), our ${D}_{{{{{\rm{N}}}}}}^{{{{{\rm{metal}}}}}/{{{{{\rm{silicate}}}}}}}$ are consistent with previous data, also indicating equilibrium partitioning of N in our experiments.

### Model N-partitioning and -isotopic fractionation during Earth’s accretion and core-formation

Using the multistage core-formation model with inputs of the Grand Tack N-body simulation results^15,49^, and using our Eqs. (1) and (2), we modeled the N-partitioning and -isotopic fractionation between Earth’s core and mantle. We first considered Earth accreted its first 60% mass through the collisions of reduced, EC-like impactors, and then its last 40% mass through the collisions of increasingly oxidized impactors. Reduced, EC-like impactors formed at heliocentric distances of <0.9–1.2 AU with δ^15^N = −30‰, while increasingly oxidized impactors originated from great heliocentric distances (1.2–3 AU). Since the solar system δ^15^N increases with the heliocentric distance^4,20^, the increasingly oxidized impactor δ^15^N should increase from −30‰ (EC’s value) to a slightly positive value at 3 AU. We used a δ^15^N value of +5‰ for the last impactor, which added Earth’s last 10% mass through the Moon-forming giant impact, and a δ^15^N value of +5‰ represents the mixing of EC (δ^15^N = −30‰) and CC (δ^15^N = +40‰) with a same N content and mass ratio of ~1 : 1 as constrained by Mo isotopes for the Moon-forming giant impactor^19^. The N content in the impactors depends a number of conditions. For example, large and oxidized impactors may hold more N than small and reduced ones, and N is mainly stored in impactor cores^42^. For an internally differentiated planetesimal^67^, i.e., no surface magma ocean, more volatiles could be retained in the planetesimal, no matter the planetesimal is oxidized or reduced. However, even oxidized planetesimals may have undergone severe evaporative volatile loss, if the surface magma ocean lasted sufficiently long. Iron meteorites contain a few ppm to 200 ppm N^21^, whereas basalts in asteroids may contain up to 10 ppm N^68^. However, both iron meteorites and asteroid basalts may have experienced extensive volatile loss, considering the pressure-dependence of N solubility in both metallic and silicate melts^37,69^. In our model, we varied the bulk N-content from 50 ppm for small and reduced impactors to 150 ppm for large and oxidized impactors.

We also considered the other factors that potentially affect the N-content and -isotopic composition of Earth’s different reservoirs. Since N is a strong volatile element, we have to consider the degassing of the silicate magma ocean. Degassing of the silicate magma ocean may have inevitably formed a proto-atmosphere^7^. However, for Earth-sized planets, it is unlikely that the entire silicate magma ocean is in equilibrium with the proto-atmosphere in volatile partitioning^70^. We defined a factor Φ, which represents the mass percentage of silicate magma ocean that is in equilibrium with the proto-atmosphere. We fix Φ at 100% at Earth’s first ~50% accretion, but varied it from 50% to 5% at Earth’s ~50–100% accretion. In addition, the N-isotopic fractionations during degassing of the hot magma ocean are thought to be inconsiderable, as even the degassing of mid-ocean ridge basalts may have not caused N-isotopic fractionations^71^. A kinetic process during degassing of a hot silicate may cause significant N-isotope fractionation;^72^ however, it remains unfeasible to quantify such effect during the degassing of a hot magma ocean. We therefore did not consider kinetic process-induced N-isotope fractionation in our model. Hydrodynamic escape of light gases in the early atmosphere to space may have resulted in preferential loss of ^14^N in space; however, such effect on Earth’s δ^15^N is difficult to quantify and the hydrodynamic escape model is inconsistent with the abundance and isotopic composition of Earth’s atmospheric xenon^22^. We therefore did not consider hydrodynamic escape of Earth’s proto-atmosphere in our model. However, it is important to consider catastrophic loss of the Earth’s proto-atmosphere during impacting^73^, because it surely affects Earth’s total N budget. Depending on a number of parameters, such as impact velocity and angle and the impactor to target mass ratio, the loss fraction of N in the proto-atmosphere during each impacting is difficult to quantify but could vary from <5% to 100% (refs. 73,74). In the Moon-forming giant impact, ~10–50% of the growing Earth’s atmosphere could have been lost from the immediate effects of the collision^73^. We fix a N-loss fraction of 100% during Earth’s first ~50% mass accretion, and from 80% to 10% during the last ~50% accretion, because small planets more readily lose their atmosphere during impacting^74^. Finally, we considered the delivery of completely oxidized or CI chondrite-like materials from beyond 6–7 AU, which contain 1000 ppm N with δ^15^N = +40‰ (refs. 50,75), to Earth’s magma ocean after Earth accreted its 60% mass. No metal–silicate segregation occurred when CI chondrite-like material added in the magma ocean, but the N in the silicate magma ocean participated in the subsequent core-formation events when metal-bearing impactors were accreted^15^. Inefficient emulsification of impactor cores may have occurred during Earth’s accretion of large and oxidized impactors. We used the degree of core–mantle disequilibrium during accretion as that in ref. 49. We also considered the light element content of the metallic melt that segregated into Earth’s core; we used a C-content of 0.2 wt.% and S-content of 1.5 wt.% (see below), and Si- and O-contents following the model in refs. 15,49.

The *P*–*T*–*f*O_2_ conditions of metal–silicate equilibration are critical for the resulting mantle and core compositions because of the dependence of ${D}_{N}^{{{{{{\rm{metal}}}}}/{silicate}}}$ and ∆^15^ *N*^metal-silicate^ on *P*–*T*–*f*O_2_. We used the approach used by refs. 15,49 for modeling multistage core formation. For each impact-induced core formation event, the metal–silicate equilibration pressure *P*_*e*_ is a constant fraction of the target’s evolving core–mantle boundary pressure:

10 $${P}_{e}={f}_{P}\times {P}_{{{{{{\rm{CMB}}}}}}}$$

where *f*_*P*_ is a constant proportionality factor; *P*_CMB_, the core–mantle boundary (CMB) pressure at the time of impact. The metal–silicate equilibration temperature, *T*_*e*_, lies between the peridotite liquidus and solidus at the equilibration pressure *P*_*e*_. The equilibration *f*O_2_ varied from ~IW-5 to IW-2 with the accreted impactors changing from reduced to oxidized composition.

In an *i*th stage of collisional accretion, the distribution of N among the reservoirs of Earth’s core, silicate magma ocean, and atmosphere must follow a mass balance:

11 $${M}_{i}^{{{{{\rm{N}}}}}-{{{{{\rm{atm}}}}}}}+{M}_{i}^{{{{{\rm{N}}}}}-{{{{\rm{silicate}}}}}}+{M}_{i}^{{{{{\rm{N}}}}}-{{{{{\rm{metal}}}}}}}={M}_{i}^{{{{{\rm{N}}}}}-{{{{{\rm{bulk}}}}}}}$$

where ${M}_{i}^{{{{{\rm{N}}}}}-{{{{{\rm{atm}}}}}}}$, ${M}_{i}^{{{{{\rm{N}}}}}-{{{{{\rm{silicate}}}}}}}$, and ${M}_{i}^{{{{{\rm{N}}}}}-{{{{{\rm{metal}}}}}}}$ are the N mass in the atmosphere, silicate melt, and metallic melt, respectively; ${M}_{i}^{{{{{\rm{N}}}}}-{{{{{\rm{bulk}}}}}}}$ is the total N mass that participates in the equilibrium partitioning. Equation (11) can be further written as:

12 $${{M}_{i-1}^{{{{{\rm{N}}}}}-{atm}}-{M}_{i}^{{{{{\rm{N}}}}}-{{{{{\rm{los}}}}}s}}+C}_{i-1}^{{{{{\rm{N}}}}}-{{{{{\rm{silicate}}}}}}}{\times M}_{i-1}^{{{{{{\rm{silicate}}}}}}}+{C}_{i}^{{{{{\rm{N}}}}}-{{{{{\rm{imp}}}}}}}\times {M}_{i}^{{{{{{\rm{imp}}}}}}}\\={C}_{i}^{{{{{\rm{N-{silicate}}}}}}}{\times M}_{i}^{{{{{{\rm{silicate}}}}}}}+{C}_{i}^{{{{{\rm{N}}}}}-{{{{{\rm{metal}}}}}}}{\times M}_{i}^{{{{{{\rm{metal}}}}}}}+{M}_{i}^{{{{{{\rm{atm}}}}}}}$$

13 $${C}_{i}^{{{{{\rm{N}}}}}-{{{{{\rm{metal}}}}}}}={D}_{{{{{\rm{N}}}}}(i)}^{{{{{{\rm{metal}}}}}}/{{{{{\rm{silicate}}}}}}}\times {C}_{i}^{{{{{\rm{N}}}}}-{{{{{\rm{silicate}}}}}}}$$

In Eqs. (12) and (13), ${M}_{i}^{{{{{\rm{N}}}}}-{{{{{\rm{loss}}}}}}}$ denotes the mass of N lost during impacting; ${C}_{i-1}^{{{{{\rm{N}}}}}-{{{{{\rm{silicate}}}}}}}$ denotes the N concentration in the silicate melt at the *(i-*1*)*th stage of accretion; ${M}_{i-1}^{{{{{{\rm{silicate}}}}}}}$ denotes the mass of silicate melt at the *(i-*1*)*th stage of accretion; ${C}_{i}^{{{{{\rm{N}}}}}-{{{{{\rm{imp}}}}}}}$ denotes the N concentration in the impactor; ${M}_{i}^{{{{{{\rm{imp}}}}}}}$ is the mass of the impactor; ${C}_{i}^{{{{{\rm{N}}}}}-{{{{{\rm{metal}}}}}}}$ and ${C}_{i}^{{{{{\rm{N}}}}}-{{{{{\rm{silica}}}}}{te}}}$ denote the N concentration in the metallic and silicate melts, respectively. ${D}_{{{{{\rm{N}}}}}(i)}^{{{{{{\rm{metal}}}}}}/{{{{{\rm{silicate}}}}}}}$ are a function of pressure, temperature, *f*O_2_, and compositions of the metallic and silicate melts, as summarized in Eq. (1) in the main text. In order to model the N partitioning between the atmosphere and the silicate, the N solubility in silicate melt is needed. If the mantle is in full equilibrium with the atmosphere, its N concentration should be equal to the N solubility corresponding to the partial pressure of N_2_ in the atmosphere. Following the model of Libourel et al.^53^, the N solubility in silicate melt (${S}_{i}^{{{{{\rm{N}}}}}-{{{{{\rm{silicate}}}}}}},{{{{{\rm{ppm}}}}}}$) can be written as:

14 $${S}_{i}^{{{{{\rm{N}}}}}-{{{{{\rm{silicate}}}}}}}({{{{{\rm{ppm}}}}}})=0.0611\times {P}_{{{{{\rm{N}}}}}}+5.97\times {10}^{-10}\times {{{f{{{{\rm{O}}}}}}}_{2}}^{-\frac{3}{4}}\times {{P}_{{{{{\rm{N}}}}}}}^{1/2}$$

where *P*_N_ is the partial pressure of N in the atmosphere. Generally, the partial pressure of a volatile element *E* (*P*_*E*_) on the surface of magma ocean can be expressed as:

15 $${P}_{E}=r\times {M}_{i}^{{{{{{\rm{atm}}}}}}}\times {g}_{i}/{A}_{i}^{{{{{{\rm{surf}}}}}}}$$

where *g*_*i*_ is the gravitational acceleration, ${A}_{i}^{{{{{{\rm{surf}}}}}}}$ is surface area of the planet, and *r* is the mass number ratio of the volatile species and the element of interest (for N_2_, *r* = 1). It is worth noting that in Eq. (14), the *f*O_2_ denotes to the *f*O_2_ of the surface magma ocean. Following the method of ref. 76, we calculated the surface magma ocean *f*O_2_, which varies from IW−3.6 to IW+3.0 during Earth’s early to late accretions in our model. The *f*O_2_ variation could significantly affect the composition of the proto-atmosphere^77^. The combination of Eqs. (12)–(15) can be used to constrain the N distribution in the atmosphere, silicate magma ocean, and core in the framework of a multistage core-formation model.

For N-isotopic fractionations among the atmosphere, silicate melt, and metallic melt at the *i*th stage of accretion, δ^15^N also follows the mass balance rule:

16 $${{{{{{\rm{\delta }}}}}}}^{15}{{{{{\rm{N}}}}}}_{i}^{{{{{{\rm{silicate}}}}}}}\times f{{{{{\rm{N}}}}}}_{i}^{{{{{{\rm{silicate}}}}}}}+{{{{{{\rm{\delta }}}}}}}^{15}{{{{{\rm{N}}}}}}_{i}^{{{{{{\rm{metal}}}}}}}\times f{{{{{\rm{N}}}}}}_{i}^{{{{{{\rm{metal}}}}}}}+{{{{{{\rm{\delta }}}}}}}^{15}{{{{{\rm{N}}}}}}_{i}^{{{{{{\rm{atm}}}}}}}\times f{{{{{\rm{N}}}}}}_{i}^{{{{{{\rm{atm}}}}}}}={{{{{{\rm{\delta }}}}}}}^{15}{{{{{\rm{N}}}}}}_{i}^{{{{{{\rm{bulk}}}}}}}$$

where $f{{{{{\rm{N}}}}}}_{i}^{{{{{{\rm{silicate}}}}}}}$, $f{{{{{\rm{N}}}}}}_{i}^{{{{{{\rm{metal}}}}}}}$, and $f{{{{{\rm{N}}}}}}_{i}^{{{{{{\rm{atm}}}}}}}$ are the N fractions in the silicate melt, metallic melt, and atmosphere, respectively; ${{{{{{\rm{\delta }}}}}}}^{15}{{{{{\rm{N}}}}}}_{i}^{{{{{{\rm{bulk}}}}}}}$ is the bulk N isotope composition of the silicate melt, metallic melt, and atmosphere. At the *i*th stage of accretion, ${{{{{{\rm{\delta }}}}}}}^{15}{{{{{\rm{N}}}}}}_{i}^{{{{{{\rm{bulk}}}}}}}$ can be expressed as:

17 $$	 {{{{{{\rm{\delta }}}}}}}^{15}{{{{{\rm{N}}}}}}_{i}^{{{{{{\rm{bulk}}}}}}}={{{{{{\rm{\delta }}}}}}}^{15}{{{{{\rm{N}}}}}}_{i-1}^{{{{{{\rm{silicate}}}}}}}{\times} \left({C}_{i-1}^{{{{{\rm{N}}}}}-{{{{{\rm{silicate}}}}}}}{\times M}_{i-1}^{{{{{{\rm{silicate}}}}}}}\right)/\\ 	 \left({C}_{i-1}^{{{{{{\rm{N}}}}}}-{{{{{\rm{silicate}}}}}}}{\times M}_{i-1}^{{{{{{\rm{silicate}}}}}}}+{C}_{0}^{{{{{{\rm{N}}}}}}-{{{{{\rm{imp}}}}}}}\times {M}_{i}^{{{{{{\rm{imp}}}}}}}+{M}_{i-1}^{{{{{{\rm{N}}}}}}-{{{{{\rm{atm}}}}}}}-{M}_{i}^{{{{{{\rm{N}}}}}}-{{{{{\rm{loss}}}}}}}\right)\\ 	+{{{{{{{\rm{\delta }}}}}}}^{15}{{{{{\rm{N}}}}}}_{i}^{{{{{{\rm{imp}}}}}}}\times C}_{i}^{{{{{{\rm{N}}}}}}-{{{{{\rm{imp}}}}}}}\times {M}_{i}^{{{{{{\rm{imp}}}}}}}/\Big({C}_{i-1}^{{{{{{\rm{N}}}}}}-{{{{{\rm{silicate}}}}}}}{\times M}_{i-1}^{{{{{{\rm{silicate}}}}}}}\\ 	+{C}_{0}^{{{{{{\rm{N}}}}}}-{{{{{\rm{imp}}}}}}}\times {M}_{i}^{{{{{{\rm{imp}}}}}}}+{M}_{i-1}^{{{{{{\rm{N}}}}}}-{{{{{\rm{atm}}}}}}}-{M}_{i}^{{{{{{\rm{N}}}}}}-{{{{{\rm{loss}}}}}}}\Big)\\ 	+{{{{{{{\rm{\delta }}}}}}}^{15}{{{{{\rm{N}}}}}}_{i-1}^{{{{{{\rm{atm}}}}}}}\times} \left({M}_{i-1}^{{{{{{\rm{N}}}}}}-{{{{{\rm{atm}}}}}}}-{M}_{i}^{{{{{{\rm{N}}}}}}-{{{{{\rm{loss}}}}}}}\right)/\\ 	 \left({C}_{i-1}^{{{{{{\rm{N}}}}}}-{{{{{\rm{silicate}}}}}}}{\times M}_{i-1}^{{{{{{\rm{silicate}}}}}}}+{C}_{0}^{{{{{{\rm{N}}}}}}-{{{{{\rm{imp}}}}}}}\times {M}_{i}^{{{{{{\rm{imp}}}}}}}+{M}_{i-1}^{{{{{{\rm{N}}}}}}-{atm}}-{M}_{i}^{{{{{{\rm{N}}}}}}-{{{{{\rm{loss}}}}}}}\right)$$

where ${{{{{{\rm{\delta }}}}}}}^{15}{{{{{\rm{N}}}}}}_{i}^{{{{{{\rm{imp}}}}}}}$ is the N-isotopic composition of the accreting impactor. The N-isotopic fractionation between metallic melt and silicate melt is:

18 $${{{{{{\rm{\delta }}}}}}}^{15}{{{{{\rm{N}}}}}}_{i}^{{{{{{\rm{metal}}}}}}}{-{{{{{\rm{\delta }}}}}}}^{15}{{{{{\rm{N}}}}}}_{i}^{{{{{{\rm{silicate}}}}}}}={\triangle }^{15}{{{{{\rm{N}}}}}}_{i}^{{{{{{\rm{metal}}}}}}-{{{{{\rm{silicate}}}}}}}$$

${\triangle }^{15}{{{{{\rm{N}}}}}}_{i}^{{{{{{\rm{metal}}}}}}-{{{{{\rm{silicate}}}}}}}$ is a multi-function of temperature and *f*O_2_, as summarized in Eq. (2) in the main text. The equilibrium N-isotopic fractionation between the atmosphere and silicate melt at high temperatures is negligible^71^, so the following equation was used:

19 $${{{{{{\rm{\delta }}}}}}}^{15}{{{{{\rm{N}}}}}}_{i}^{{{{{{\rm{atm}}}}}}}{-{{{{{\rm{\delta }}}}}}}^{15}{{{{{\rm{N}}}}}}_{i}^{{{{{{\rm{silicate}}}}}}}={\triangle }^{15}{{{{{\rm{N}}}}}}_{i}^{{{{{{\rm{atm}}}}}}-{{{{{\rm{silicate}}}}}}}=0$$

The combination of Eqs. (16)–(19) can be used to constrain the N-isotopic composition in the atmosphere, silicate magma ocean, and core in the framework of a multistage core formation model.

### Test the sensitivity of varying the parameters used in the above N-accretion model

We recognized that during Earth’s multistage accretion, the processes such as collision-induced catastrophic atmosphere loss and the degree of silicate magma ocean–atmosphere equilibrium remain poorly constrained. We therefore performed additional modeling to test the effect of varying the parameters used in our model on the final N-content and δ^15^N of the proto-Earth’s atmosphere and mantle (Supplementary Figs. 8−14). Supplementary Fig. 8 shows that fixing Φ at 20–60% would result in N-content and δ^15^N of ~1.1 to 1.7 ppm and ~ −1.8‰ to −0.3‰ for the proto-atmosphere, and ~0.7 to 2.2 ppm and ~ −6.8‰ to −0.1‰ for the proto-mantle. These values cover the observed ones for Earth’s mantle and surface. Supplementary Fig. 9 shows that varying the degree of impact-induced atmospheric loss from 40% to 60% would vary the proto-atmospheric N-content from 0.7 to 1.5 ppm and δ^15^N from −3‰ to +4.2‰, but does not change the mantle values. These proto-atmospheric values also cover those observed values for Earth’s surface reservoir. In addition, Supplementary Fig. 10 shows that slightly increasing the degree of equilibrium between silicate magma ocean and overlying atmosphere or decreasing the added mass of CI chondrites would decrease the proto-atmosphere δ^15^N to ~0‰ and meanwhile increase the atmosphere N-content at ~1 ppm. Supplementary Fig. 11 shows that changing the relative timing of the delivery of CI chondrite-like materials would either not change the main conclusions, as long as the CI chondrite-like materials were delivered after Earth accreting its ~60% mass but before complete core–mantle segregation. Supplementary Fig. 12 shows that varying the C-content in the impactor core from 0.5 to 2 wt.% would either not change the conclusions. Supplementary Fig. 13 shows that varying the relative contributions of EC and CC to the Moon-forming giant impactor in δ^15^N would nearly not affect the δ^15^N of the proto-Earth’s atmosphere, mantle, and core. This is because most of the N in the last impactor was in the impactor core, and limited emulsification (5%) of the impactor core resulted in most of the N in the impactor delivered to Earth’s core. The N-content in the impactors must affect Earth’s bulk N-content. We compare the modeling results using impactors containing 80–200 ppm N (N-rich) and 20–100 ppm N (N-poor) in Supplementary Fig. 14. It shows that the final N-contents in the proto-atmosphere (1.8 vs. 1.4 ppm) and proto-mantle (2.9 vs. 2.1 ppm, respectively) are well comparable, while the N-content in the core is more different in these two cases (234 vs. 118 ppm), because N is siderophile during the whole accretion phase. Supplementary Fig. 14 also shows that the δ^15^N of the proto-atmosphere, proto-mantle, and core is nearly not changed in these two cases, and any slight change in δ^15^N can be counterbalanced by slightly increasing the mass of CI chondrites added.

### Constrain the distribution and origin of other major volatiles (C–H–S) during Earth’s heterogeneous accretion

The other major volatiles (C–H–S) would undergo similar accretion process as modeled above for N. Since these volatile elements have very different geochemical behaviors relative to N, we had to consider the C–H–S contents in the accreted materials, their solubilities in silicate melts at the atmosphere–mantle equilibrium conditions, and their metal/silicate partition coefficients at the core-formation conditions, before we applied our above N-accretion model to constrain the distribution and origin of C–H–S in Earth’s different reservoirs.

For the C–H–S contents in the accreted materials, we assumed that the completely oxidized materials have CI chondrite-like C–H–S contents, and the C–H–S contents in other impactors were inferred from the asteroid meteorites. Hydrogen is mainly concentrated in the silicate part of the impactors due to the lithophile nature of H under asteroid conditions where the interior pressure is <20 GPa. Since the H contents in the achondrites are in the range of 50–170 ppm^78,79^, in our model the bulk H contents were changed from ~50 ppm in the reduced impactors to ~150 ppm in the oxidized impactors. Carbon and S are more concentrated in the metal core of the impactors because of their siderophile nature under asteroids conditions^80,81^. The C contents in iron meteorites are no more than 1500 ppm^82^, and the C contents in achondrites are in the range of 10–140 ppm^83,84^. We hence set the C contents in the impactors at ~150–1000 ppm. The S contents vary greatly in iron meteorites (0.4–19 wt.%)^82^ and achondrites (110–5000 ppm)^85,86^. However, the log(C/S) of iron meteorites are well correlated with the log(C) (ref. 82). Hence, we set the S contents in the bulk impactors in the range of ~2000–11000 ppm.

The surface magma ocean *f*O_2_ changes from IW−3.6 to IW+3 during Earth’s accretion in our model, as stated above, which would significantly influence the species and hence the solubilities of C–H–S in silicate melts^77^. In silicate melt, the relative fraction of H existing as H_2_ or H_2_O follows a relationship as:^87^

20 $${{{{{{\rm{H}}}}}}}_{2}({{{{{\rm{wt}}}}}}.\%)=0.0873\times {{{{{{\rm{H}}}}}}}_{2}{{{{{\rm{O}}}}}}({{{{{\rm{wt}}}}}}.\%) - 0.0044\times \Delta {{{{{\rm{IW}}}}}} - 0.0198$$

In our model, the H contents in the impactors are no more than 150 ppm (see above), Eq. (20) indicates that the fraction of H as H_2_ is less than 30% at *f*O_2_ of IW−3.6 and would be negligible if the *f*O_2_ is higher than IW−2.5. This means that the main H species in the surface magma ocean is H_2_O during the whole accretion process. We hence used the H_2_O solubility model from ref. 88 in our model:

21 $$2{ln}{X}^{{{{{{\rm{H}}}}}}_{2}{{{{\rm{O}}}}}-{{{{{\rm{silicate}}}}}}}=\frac{2565}{T}+\sum {b}_{j}{X}_{j}\frac{{P}_{{{{{\rm{g{as}}}}}}}}{T}+1.171\times {ln}{f{{{{{{\rm{H}}}}}}}_{2}{{{{{\rm{O}}}}}}}^{{{{{{\rm{fluid}}}}}}}-14.21$$

where ${X}^{{{{{{\rm{H}}}}}}_{2}{{{{{\rm{O}}}}}}-{{{{{\rm{silicate}}}}}}}$ and ${f{{{{{{{\rm{H}}}}}}}}_{2}{{{{{\rm{O}}}}}}}^{{{{{{\rm{fluid}}}}}}}$ are the mole fraction of water in the silicate melt and the fugacity of water in the fluid, respectively. *P*_gas_ is the total pressure of the gas, *b*_*j*_ and *X*_*j*_ are the constant factor and oxide mole fraction of component *j* in silicate melt (*b*_Al2O3_ = −1.997, *b*_FeO(T)_ =  −0.9275, *b*_Na2O_ = 2.376)^56^. Assuming that the fugacity coefficient of H_2_O is equal to 1, the ${f{{{{{{\rm{H}}}}}}}_{2}{{{{{\rm{O}}}}}}}^{{fluid}}$ is equal to its partial pressure and could be calculated by using Eq. (15) with *r*(H_2_O) = 9. The *P*_gas_ could be obtained by summing the partial pressure of all volatiles in the atmosphere.

The species of C in H-bearing silicate melts are also controlled by *f*O_2_. At reduced conditions (*f*O_2_ < IW−1), the main C species is methane, while at oxidized conditions (*f*O_2_ >IW−1), the main C species is carbonate^89^. For C solubility in silicate melt (*S*^*C-*silicate^) at *f*O_2_ < IW−1, we used the model from ref. 89 as:

22 $${{\log }}\,{S}^{C-{{{{{\rm{silicate}}}}}}}({{{{{\rm{ppm}}}}}})=0.96\times {{{\log }}X}^{{{{{{\rm{H2O}}}}}}-{{{{{\rm{silicate}}}}}}} - 0.25\times \Delta {{{{{\rm{IW}}}}}}+2.83$$

At *f*O_2_ >IW−1, we used the model from ref. 90, which is more valid for peridotitic melt. For S solubility in silicate melt (*S*^*S-*silicate^), we used a recent model of ref. 77:

23 $${{{{{\rm{ln}}}}}}\,{S}^{S-{{{{{\rm{silicate}}}}}}}(ppm)=13.8426 - 26476/T({{{{{\rm{K}}}}}})+0.124\times {C}^{{{{{{\rm{FeO}}}}}}}+0.5\times {{{{{\rm{ln}}}}}}({f{{{{{\rm{S}}}}}}}_{2}/{f{{{{{\rm{O}}}}}}}_{2})$$

where the *C*^FeO^ is the FeO content in the silicate melt (in wt.%), *f*S_2_ is sulfur fugacity, which could be approximately regarded as partial pressure of S_2_ in the atmosphere, and then could be expressed in a form like Eq. (15).

As for the metal/silicate partition coefficients, we used the parameterized ${D}_{H(i)}^{{{{{{{\rm{metal}}}}}}}/{{{{{{\rm{silicate}}}}}}}}$ from ref. 91, ${D}_{C(i)}^{{{{{{\rm{metal}}}}}}/{{{{{\rm{silicate}}}}}}}$ from ref. 80, and ${D}_{S(i)}^{{{{{{\rm{metal}}}}}}/{{{{{\rm{silicate}}}}}}}$ from ref. 43.

With the above parameters for C–H–S, we applied our N-accretion model to C–H–S to constrain the distribution of C–H–S in Earth’s different reservoirs during Earth’s heterogeneous accretion. Our results presented in Figs. 5, 6 in the main text show that our modeled C–H–S contents in Earth’s different reservoirs are consistent with the present-day observations. Our study suggests that Earth acquired its volatiles through very complicated processes, but these complicate processes happened naturally during Earth’s main accretion phase. Therefore, the establishment of Earth’s volatile inventory was a natural outcome of Earth’s heterogeneous accretion processes.

## Supplementary information

Supplementary Information

Description of Additional Supplementary Files

Supplementary Data 1

Supplementary Data 2

Supplementary Data 3

Supplementary Data 4

Supplementary Data 5

Supplementary Data 6

## Data Availability

All data supporting the findings of this study are available within the paper and supplementary information and source data files (Supplementary Data 1–6). Additional data related to this paper may be requested from the authors.
