# Supplementary material for: Nitrogen isotope evidence for Earth’s heterogeneous accretion of volatiles
Source: Nat Commun. 2022 Aug 15;13:4769. doi: 10.1038/s41467-022-32516-5 (PMC9378614; doi:10.1038/s41467-022-32516-5)
Supplement: Supplementary file 1 — Supplementary Information [file 41467_2022_32516_MOESM1_ESM.pdf]

Supplementary Information for

Nitrogen isotope evidence for Earth's heterogeneous accretion of volatiles

Lanlan Shi[1,2,3#], Wenhua Lu[1,2,3#], Takanori Kagoshima[4], Yuji Sano[4,5], Zenghao Gao[1,2,3], Zhixue Du[1,2], Yun Liu[6], Yingwei Fei[7], Yuan Li[1,2*]

[1]State Key Laboratory of Isotope Geochemistry, Guangzhou Institute of Geochemistry, Chinese Academy of Sciences, Guangzhou 510640, China

[2]CAS Center for Excellence in Deep Earth Science, Guangzhou 510640, China

[3]College of Earth and Planetary Sciences, University of Chinese Academy of Sciences, Beijing 100049, China

[4]Division of Ocean-Earth System Science, Atmosphere and Ocean Research Institute, University of Tokyo, Kashiwa, Chiba 277-8564, Japan

[5]Center for Advanced Marine Core Research, Kochi University, Nanokoku, Kochi 783-8502, Japan.

[6]International Center for Planetary Science, College of Earth Sciences, Chengdu University of Technology, Chengdu 610059, China

[7]Earth and Planets Laboratory, Carnegie Institution for Science, Washington DC 20015, USA

[#]These authors contributed equally.
*Corresponding author. E-mail address: Yuan.Li@gig.ac.cn

This PDF file includes:

1. **Supplementary Figs. 1–14**
2. **Supplementary Note 1. The references cited for the $\delta^{15}N$ data plotted in Fig. 1.**
3. **Supplementary references**

1. Supplementary Figs. 1–14:

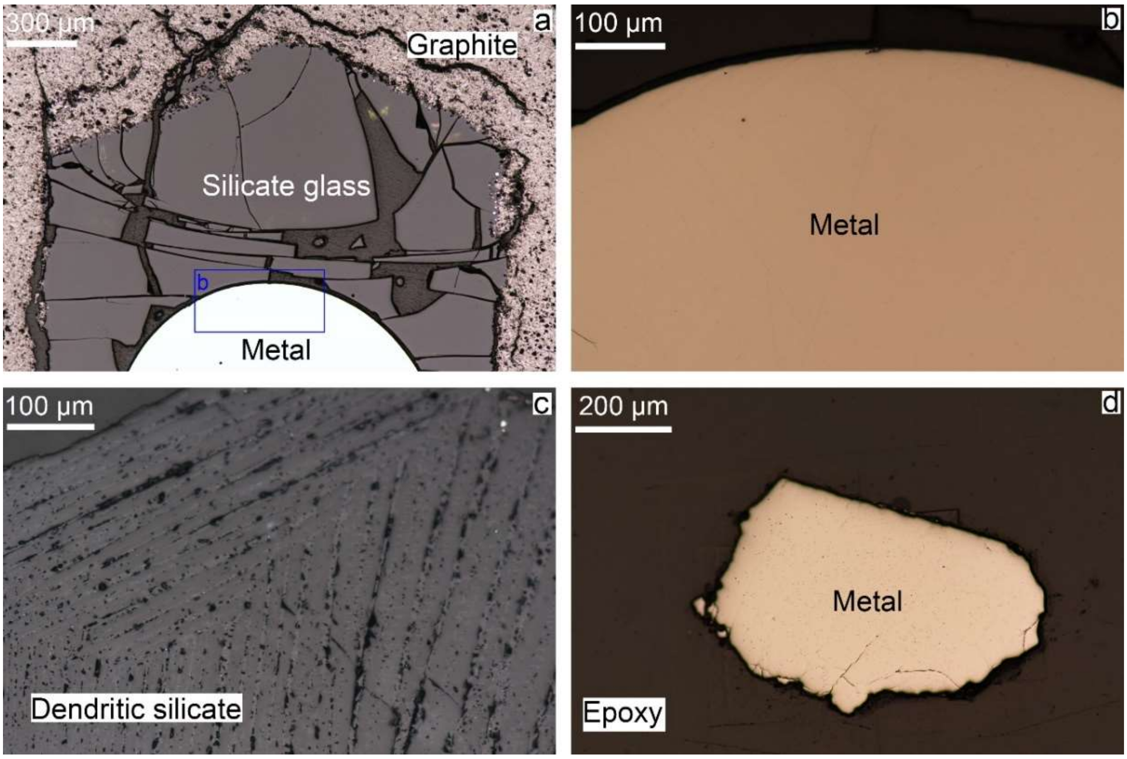

Supplementary Figure 1. Reflected-light photomicrographs of typical run products synthesized in graphite-lined $Pt_{95}Rh_{05}$ or graphite capsules. a Coexisting metallic melt and silicate melt synthesized at 1.5 GPa and 1700 °C using silicate MORB (run N-1) in a graphite-lined $Pt_{95}Rh_{05}$ capsule. The silicate melt was quenched into glass. **b** Close-up view of the quenched metallic melt in (a). **c, d** The separated metallic melt and silicate melt from run PY-2 synthesized at 8 GPa and 2000 °C using silicate pyrolite in a graphite capsule. The pyrolite melt had a typic dendritic quench texture.

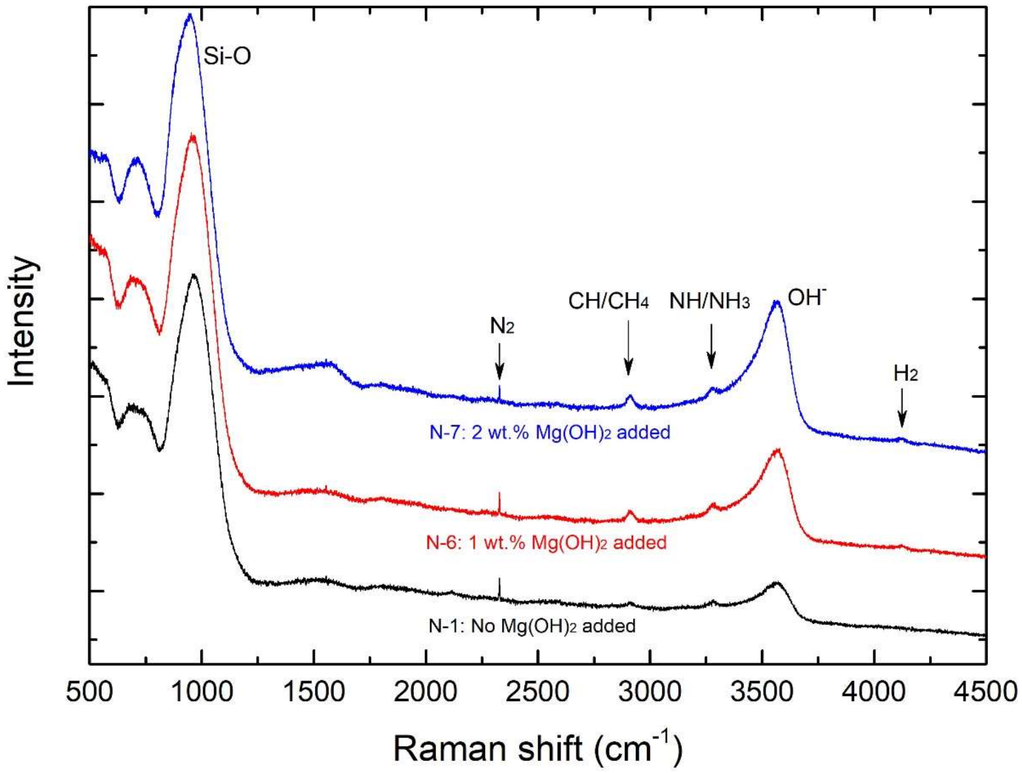

Supplementary Figure 2. Typical Raman spectral of silicate glasses from this study showing regions associated with N-C-H-O volatile species. Note that the water peak intensity increases with increasing the amount of $Mg(OH)_2$ added in the starting silicate. The experiments (N-1, N-6, and N-7; Supplementary Data 2) were synthesized at 1.5 GPa and 1700 °C, and fO_2 of ~IW-0.5. These observed N-C-H-O species are consistent with those observed in previous studies[1-4], including the one that determined the metal/silicate N-isotopic fractionations[5].

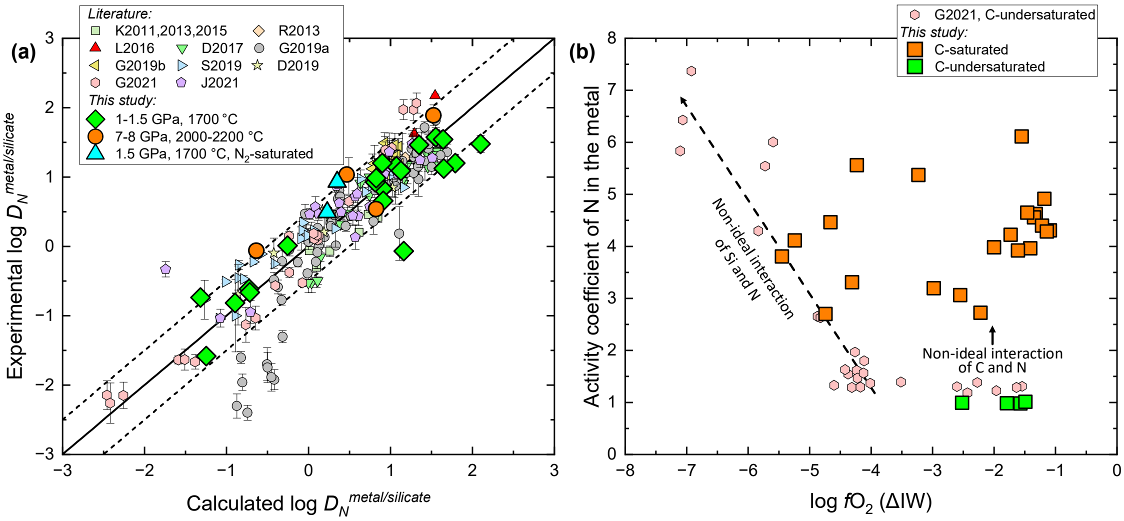

Supplementary Figure 3. a Comparison of the experimentally determined $D_N^{metal/silicate}$ with the calculated $D_N^{metal/silicate}$ using Eq. (1) in the main text. **b** The plot of activity coefficient of N in the metallic melt as a function of oxygen fugacity (ΔIW). Note the non-ideal interaction of C and N.

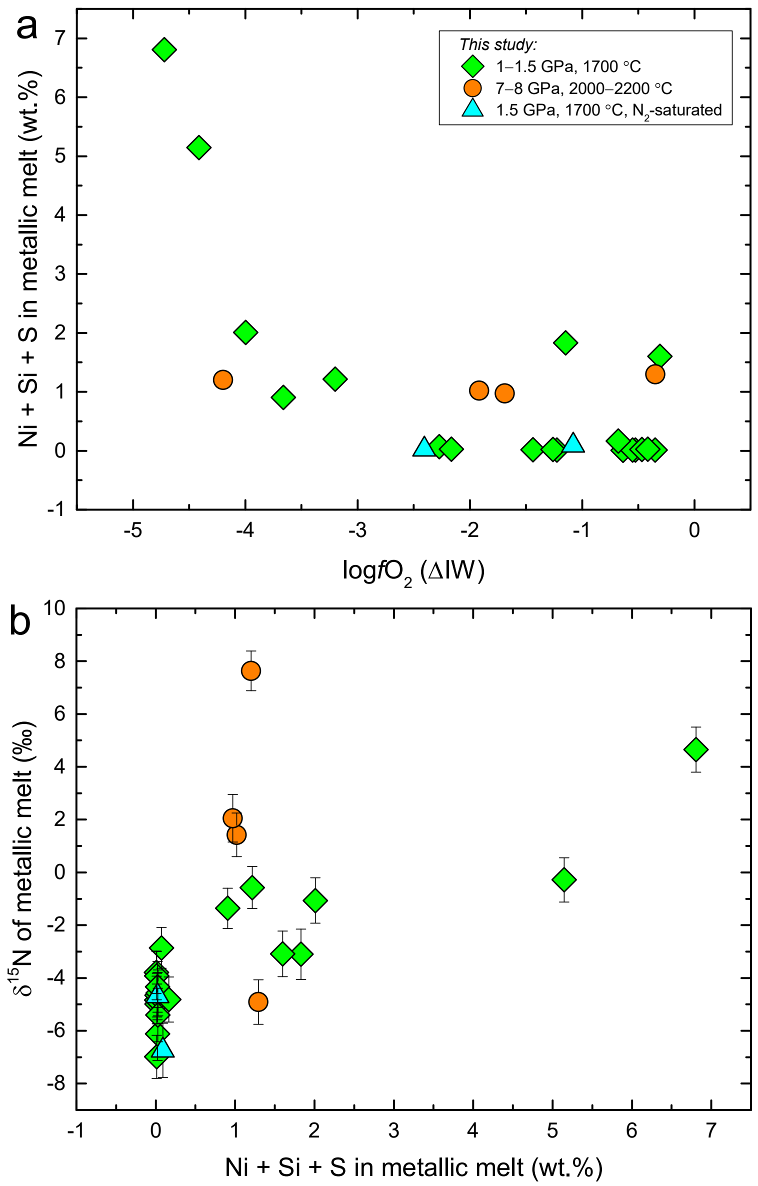

Supplementary Figure 4. Weak correlations occur between sample fO_2, the light element and Ni content in metallic melt, and the metallic melt $\delta^{15}N$. a The Ni + Si + S content in the metallic melt is in a weak negative correlation with fO_2. **b** The Ni + Si + S content in the metallic melt is in a weak positive correlation with $\delta^{15}N$ of the metallic melt.

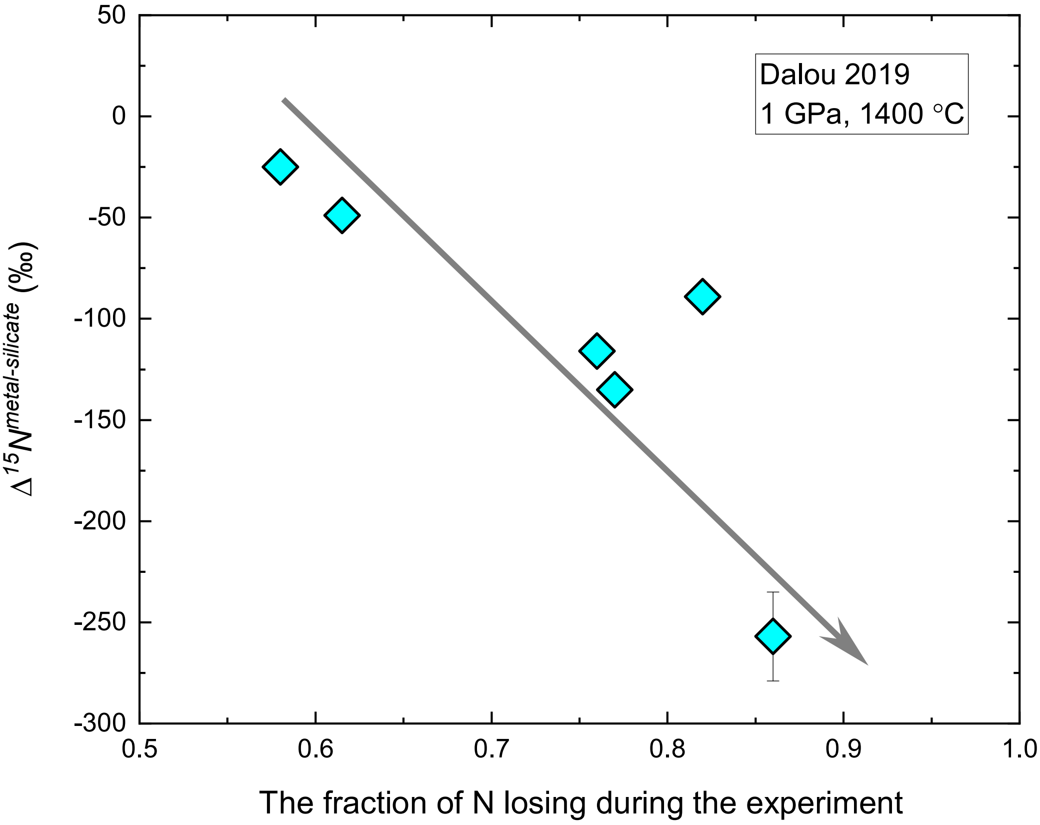

Supplementary Figure 5. The negative correlation between $\Delta^{15}N^{metal-silicate}$ and the fraction of N losing during the experiment. The data were taken from Dalou et al.[5]. The large $\Delta^{15}N^{metal-silicat}$ values are likely caused by kinetic processes, rather than representing equilibrium N-isotopic fractionations. See main text for more detailed explanations.

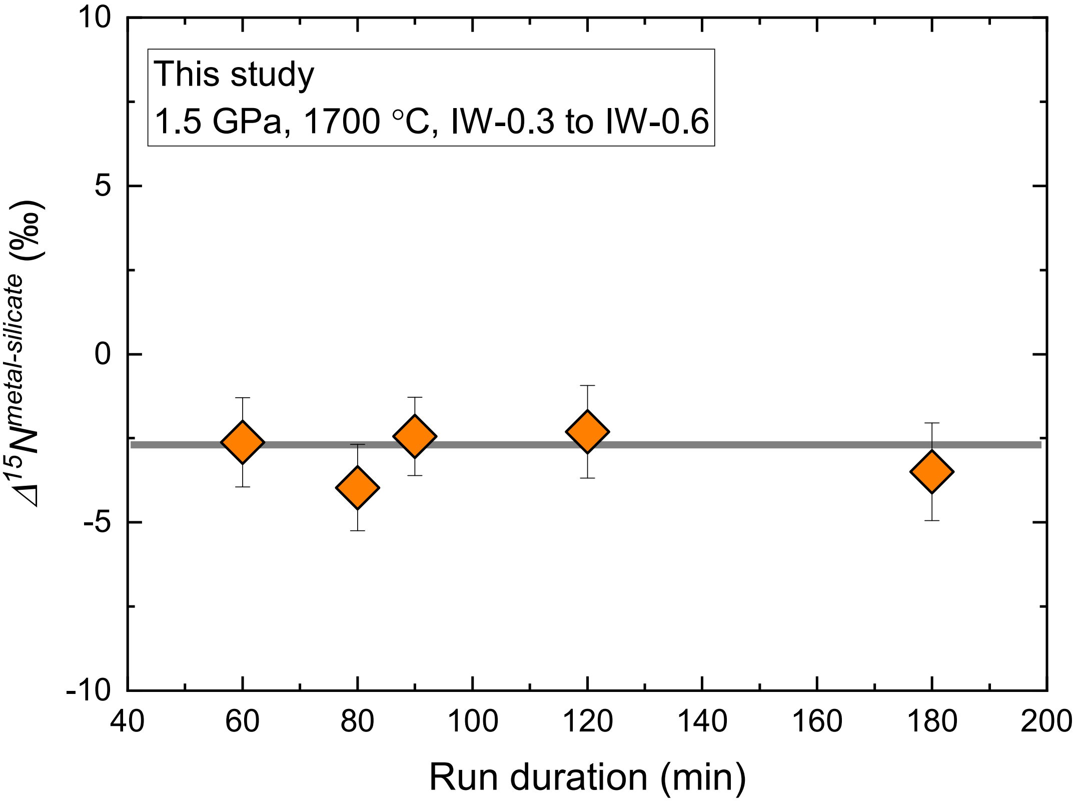

Supplementary Figure 6. The metal/silicate N-isotopic fractionation ($\Delta^{15}N^{metal-silicate}$) as a function of experimental duration. The independence of $\Delta^{15}N^{metal-silicate}$ on the experimental duration indicates that 60 mins was sufficient for approaching equilibrium at 1700 °C.

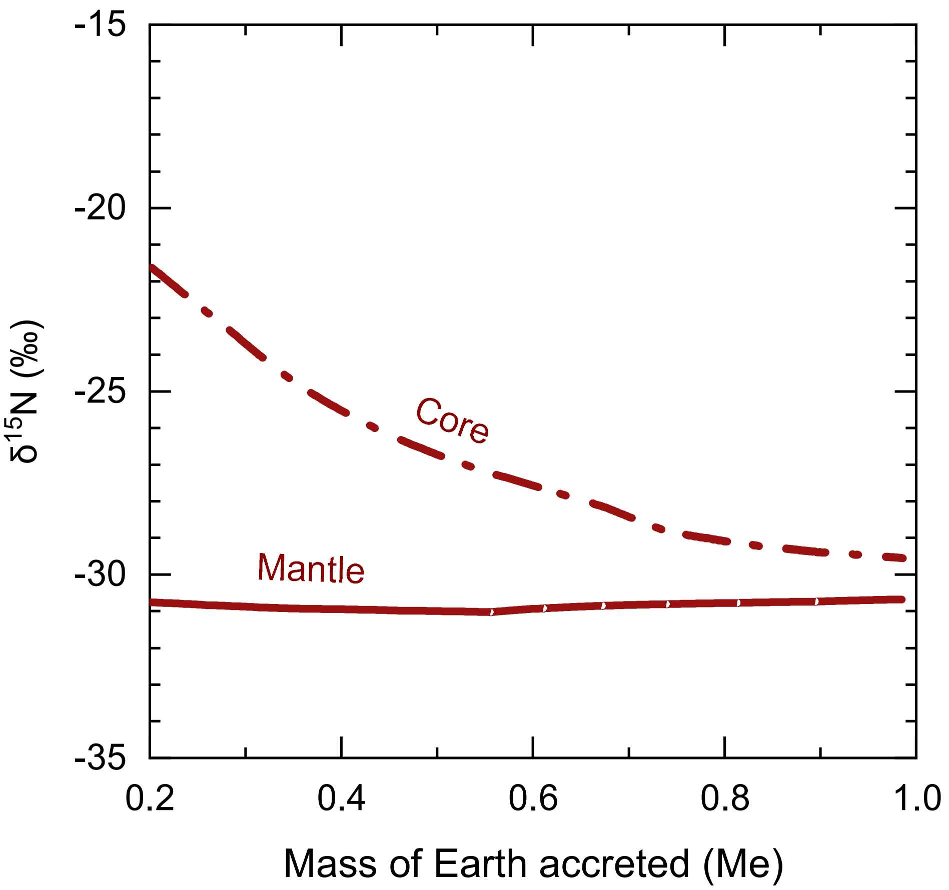

Supplementary Figure 7. Modeling N-isotopic composition of Earth's core and mantle assuming that Earth accreted its 100% mass through the collisions of differentiated impactors that have a $\delta^{15}N$ value of −30‰. The results show that core-formation alone cannot lead to a $\delta^{15}N$ value of −5‰ of Earth's present-day mantle from a starting $\delta^{15}N$ value of −30‰.

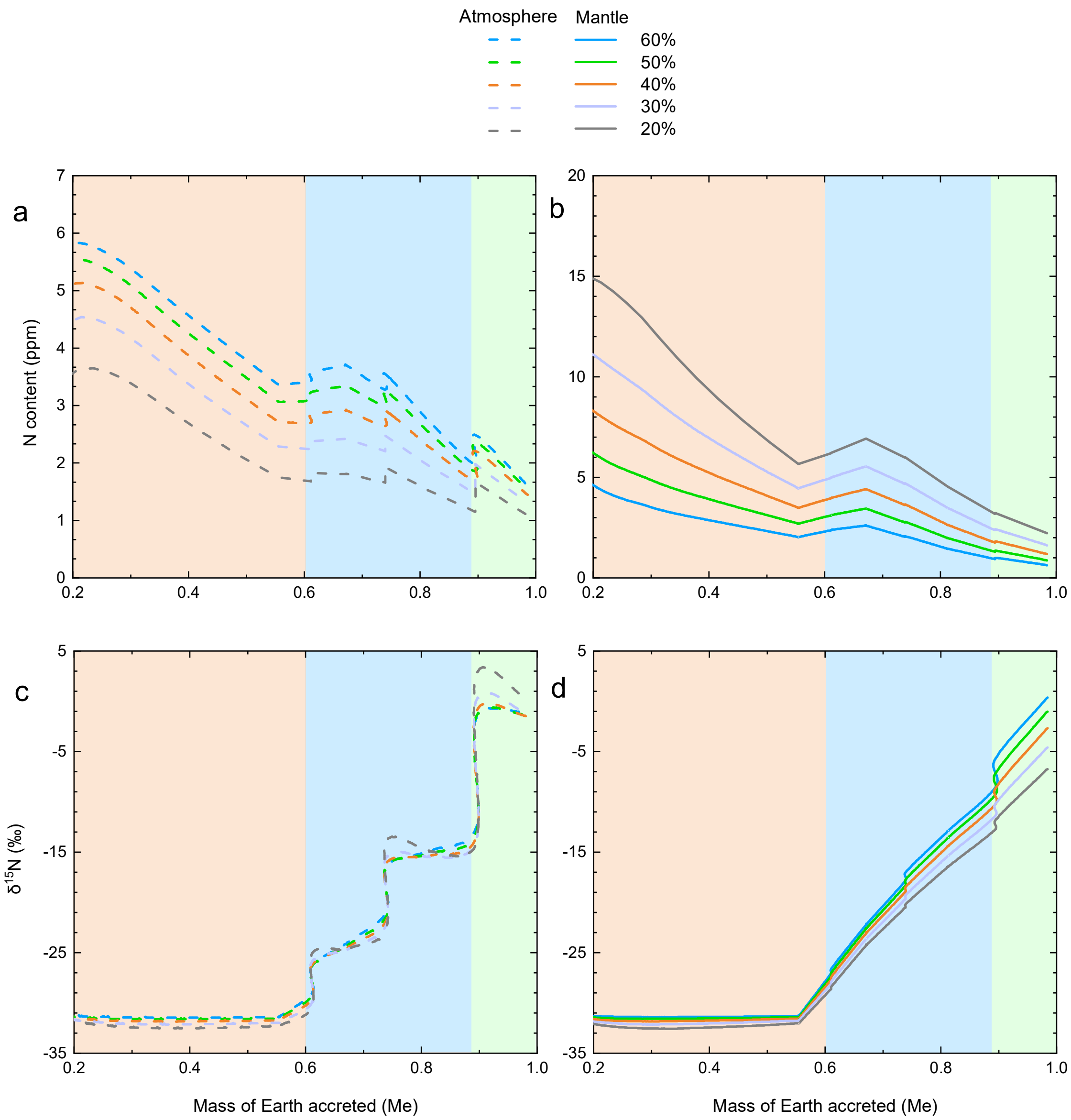

Supplementary Figure 8. The N-content and δ^{15}N of the proto-Earth's atmosphere and mantle as a function of mass accreted based on a combined model of Earth's accretion and differentiation[6,7]. a, b The N-content of the proto-Earth's atmosphere and mantle. The N-content of the atmosphere is relative (normalized) to the mass of the silicate Earth. **c, d** The δ^{15}N of the proto-Earth's atmosphere and mantle. This figure shows the effect of varying the mass percentage (Φ=20–60%) of the silicate magma ocean that is in equilibrium with the overlying atmosphere on the N-content and -isotopic composition of the proto-Earth's atmosphere and mantle. The degree of impact-induced atmosphere loss was fixed at 45% throughout all accretion stages.

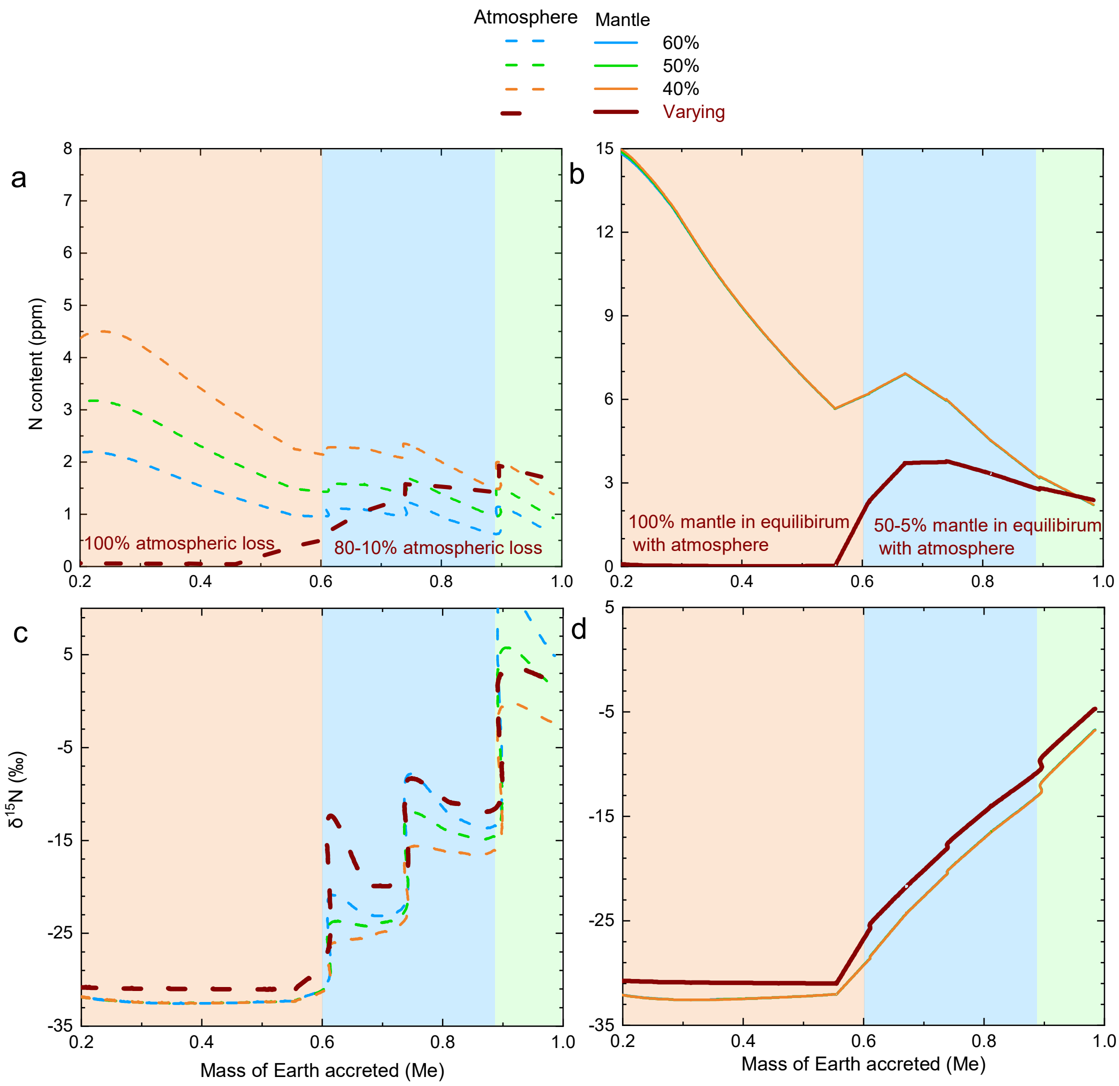

Supplementary Figure 9. The N-content and $\delta^{15}N$ of the proto-Earth's atmosphere and silicate mantle as a function of mass accreted. a, b The N-content of the proto-Earth's atmosphere and mantle. The N-content of the atmosphere is relative (normalized) to the mass of the silicate Earth. **c, d** The $\delta^{15}N$ of the proto-Earth's atmosphere and mantle. This figure shows that constant impact-induced atmosphere loss (varying from 40% to 60%) in each impact only affects the N-content and -isotopic composition of the atmosphere, without affecting those of the mantle. Φ was fixed at 20% throughout all accretion stages. Note that the case of varying the degree of atmosphere loss (deep brown curves), as presented in Fig. 4 in the main text, was also plotted for comparison.

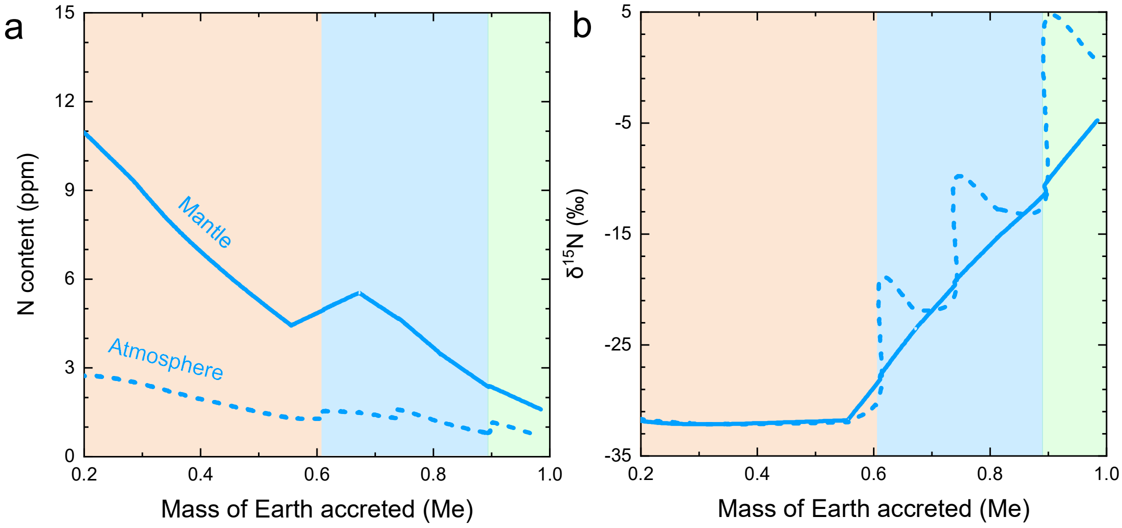

Supplementary Figure 10. Modeling results of the case with 60% impact-induced atmosphere loss (bule curve in Supplementary Fig. 9) after changing Φ from 20% to 30% and the mass of last added CI chondritic materials from 0.04% to 0.025%. a, b This figure shows that the low atmosphere N content and high atmosphere $\delta^{15}N$, caused by a high degree of impact-induced atmosphere loss as modeled in Supplementary Fig. 9 using Φ of 20%, can be increased and decreased, respectively, by changing the other parameters used in the model. The N-content of the atmosphere is relative (normalized) to the mass of the silicate Earth.

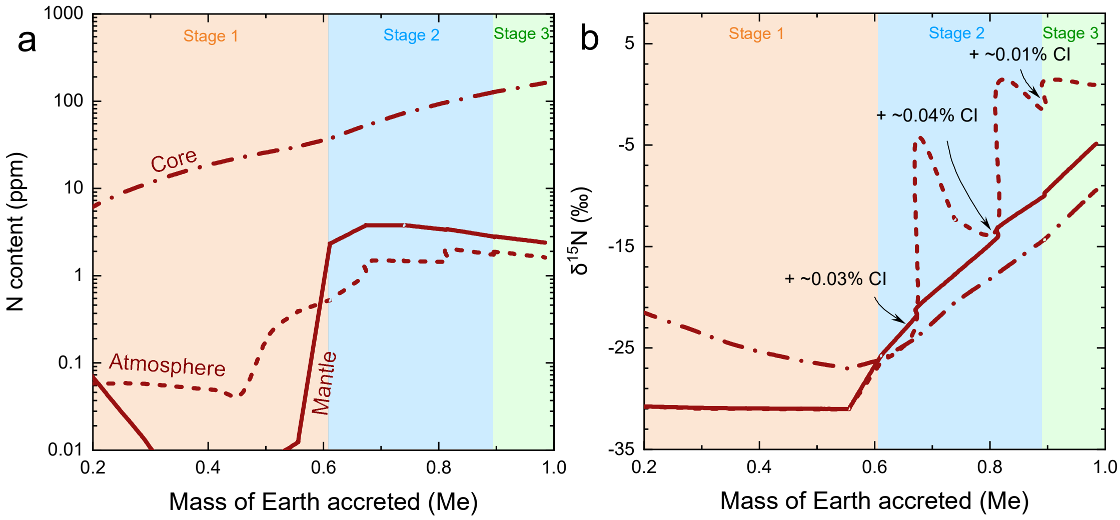

Supplementary Figure 11. The N-content and $\delta^{15}N$ of the proto-Earth's atmosphere, mantle, and core as a function of mass accreted. **a, b** This figure provides supplementary information to Fig. 4 in the main text, and shows that varying the timing of the delivery of CI chondrite-like materials would not change significantly the N-content and -isotopic composition of the proto-Earth's core, silicate mantle, and atmosphere. The N-content of the atmosphere is relative (normalized) to the mass of the silicate Earth.

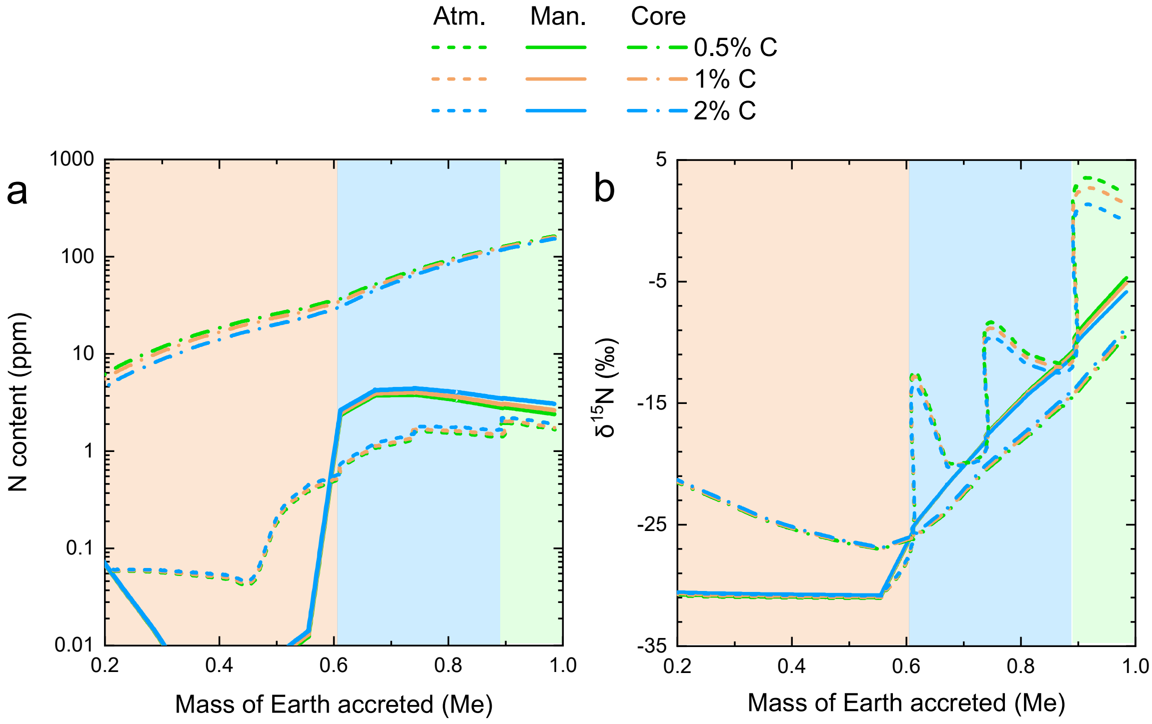

Supplementary Figure 12. The N-content and $\delta^{15}N$ of the proto-Earth's atmosphere (Atm.), silicate mantle (Man.), and core as a function of mass accreted. a, b This figure provides supplementary information to Fig. 4 in the main text, and shows that varying the C-content, from 0.5 to 2 wt.%, in Earth's core would not change significantly the N-content and -isotopic composition of the proto-Earth's atmosphere, silicate mantle, and core. The N-content of the atmosphere is relative (normalized) to the mass of the silicate Earth.

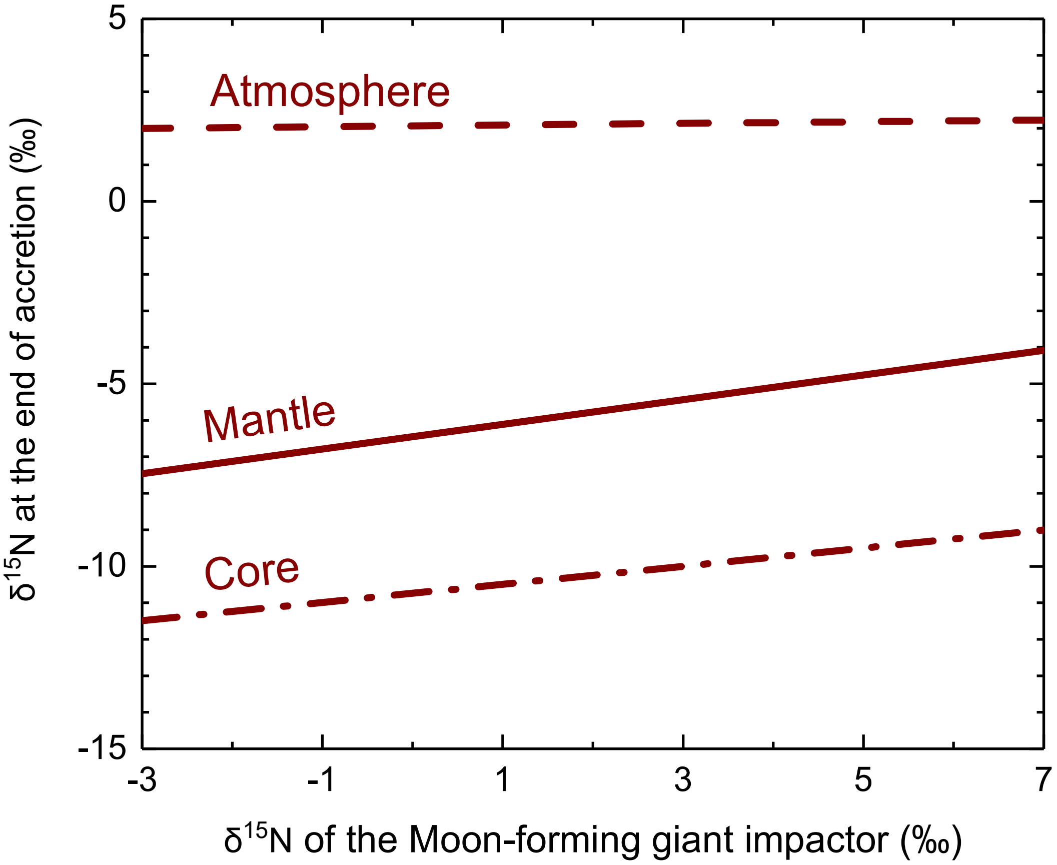

Supplementary Figure 13. Effect of varying the δ^{15}N of the Moon-forming giant impactor on the δ^{15}N of the proto-Earth's atmosphere, mantle, and core at the end of accretion. This figure shows a limited effect of the Moon-forming giant impactor on the proto-Earth δ^{15}N.

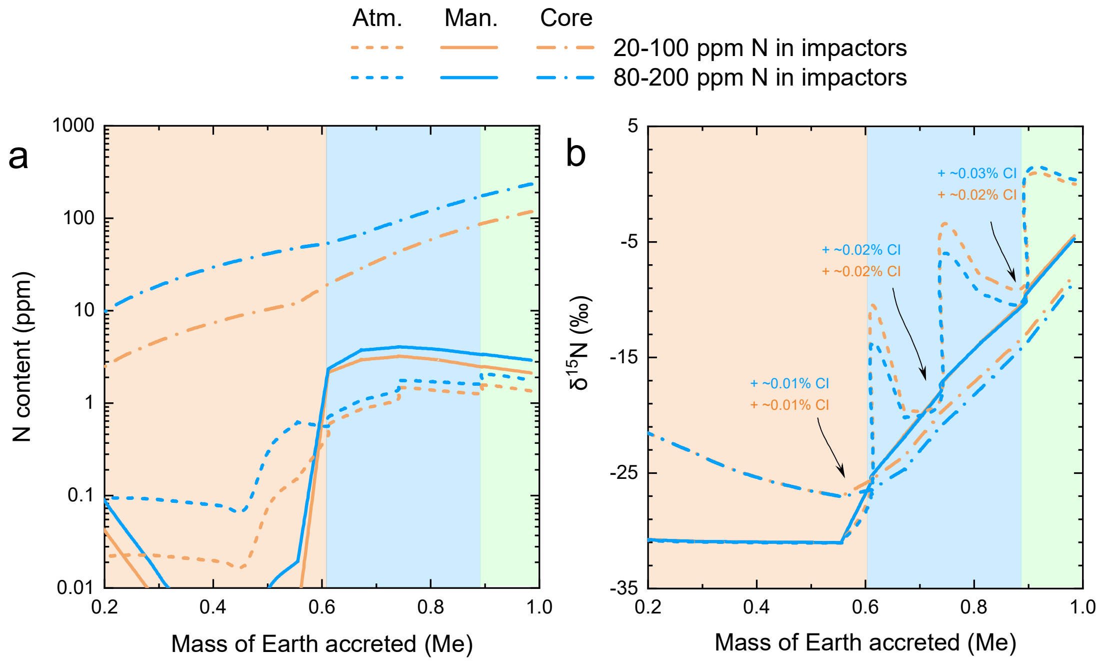

Supplementary Figure 14. The N-content and $\delta^{15}N$ of the proto-Earth's atmosphere (Atm.), silicate mantle (Man.), and core as a function of mass accreted. a, b This figure shows that the N-content in the impactors varying from 20 to 100 ppm or from 80 to 200 ppm could also explain the N-content and $\delta^{15}N$ of the proto-Earth's atmosphere and silicate mantle by slightly modifying the mass of CI-chondritic materials delivered, but the N-content in Earth's core increases clearly with increasing the impactor N content. The N-content of the atmosphere is relative (normalized) to the mass of the silicate Earth.

2. Supplementary Note 1. The references cited for the $\delta^{15}N$ data plotted in Fig. 1.

In Fig. 1, we used ref. [8] for Earth's mantle, refs. [9-11] for mantle peridotite diamonds, ref. [12] for solar wind (SW), ref. [13] for comets, refs. [14-19] for OIB, refs. [8,20] for average $\delta^{15}N$ values of modern and Archean sediments and Earth's crust, refs. [21,22] for enstatite chondrites (EC), and refs. [23-33] for the $\delta^{15}N$ values of OC, CI, CM, CR, CV, and CO chondrites.

3. Supplementary references:

1 Armstrong, L. S., Hirschmann, M. M., Stanley, B. D., Falksen, E. G. & Jacobsen, S. D. Speciation and solubility of reduced C–O–H–N volatiles in mafic melt: Implications for volcanism, atmospheric evolution, and deep volatile cycles in the terrestrial planets. *Geochimica et Cosmochimica Acta* **171**, 283-302, doi:10.1016/j.gca.2015.07.007 (2015).

2 Kadik, A. A. *et al.* Solution behavior of reduced N–H–O volatiles in FeO–Na2O–SiO2–Al2O3 melt equilibrated with molten Fe alloy at high pressure and temperature. *Phys Earth Planet In* **214**, 14-24, doi:10.1016/j.pepi.2012.10.013 (2013).

3 Li, Y., Huang, R., Wiedenbeck, M. & Keppler, H. Nitrogen distribution between aqueous fluids and silicate melts. *Earth and Planetary Science Letters* **411**, 218-228 (2015).

4 Grewal, D. S., Dasgupta, R. & Farnell, A. The speciation of carbon, nitrogen, and water in magma oceans and its effect on volatile partitioning between major reservoirs of the Solar System rocky bodies. *Geochimica et Cosmochimica Acta* **280**, 281-301, doi:10.1016/j.gca.2020.04.023 (2020).

5 Dalou, C. *et al.* Redox control on nitrogen isotope fractionation during planetary core formation. *Proc Natl Acad Sci U S A* **116**, 14485-14494, doi:10.1073/pnas.1820719116 (2019).

6 Rubie, D. C. *et al.* Heterogeneous accretion, composition and core–mantle differentiation of the Earth. *Earth and Planetary Science Letters* **301**, 31-42, doi:10.1016/j.epsl.2010.11.030 (2011).

7 Rubie, D. C. *et al.* Accretion and differentiation of the terrestrial planets with implications for the compositions of early-formed Solar System bodies and accretion of water. *Icarus* **248**, 89-108, doi:10.1016/j.icarus.2014.10.015 (2015).

8 Cartigny, P. & Marty, B. Nitrogen Isotopes and Mantle Geodynamics: The Emergence of Life and the Atmosphere-Crust-Mantle Connection. *Elements* **9**, 359-366 (2013).

9 Palot, M., Cartigny, P., Harris, J. W., Kaminsky, F. V. & Stachel, T. Evidence for deep mantle convection and primordial heterogeneity from nitrogen and carbon stable isotopes in diamond. *Earth and Planetary Science Letters* **357-358**, 179-193, doi:10.1016/j.epsl.2012.09.015 (2012).

10 Cartigny, P., Boyed, S. R., Harris, J. W. & Javoy, M. Nitrogen isotopes in peridotitic diamonds from Fuxian, China: the mantle signature. *Terra Nova*, 175-179 (1997).

11 Cartigny, P. *et al.* A mantle origin for Paleoarchean peridotitic diamonds from the Panda kimberlite, Slave Craton: Evidence from 13C-, 15N- and 33,34S-stable isotope systematics. *Lithos* **112**, 852-864, doi:https://doi.org/10.1016/j.lithos.2009.06.007 (2009).

12 Marty, B., Chaussidon, M., Wiens, R., Jurewicz, A. & Burnett, D. A 15N-poor isotopic composition for the solar system as shown by Genesis solar wind samples. *Science* **332**, 1533-1536 (2011).

13 Marty, B. *et al.* Origins of volatile elements (H, C, N, noble gases) on Earth and Mars in light of recent results from the ROSETTA cometary mission. *Earth and Planetary Science Letters* **441**, 91-102, doi:10.1016/j.epsl.2016.02.031 (2016).

14 Labidi, J. *et al.* Hydrothermal (15)N(15)N abundances constrain the origins of mantle nitrogen. *Nature* **580**, 367-371, doi:10.1038/s41586-020-2173-4 (2020).

15 Marty, B. & Dauphas, N. The nitrogen record of crust–mantle interaction and mantle convection from Archean to Present. *Earth and Planetary Science Letters* **206**, 397-410, doi:10.1016/s0012-821x(02)01108-1 (2003).

16 Barry, P. H., Hilton, D. R., Halldórsson, S. A., Hahm, D. & Marti, K. High precision nitrogen isotope measurements in oceanic basalts using a static triple collection noble gas mass spectrometer. *Geochemistry, Geophysics, Geosystems* **13**, doi:10.1029/2011gc003878 (2012).

17 Halldórsson, S. A., Hilton, D. R., Barry, P. H., Füri, E. & Grönvold, K. Recycling of crustal material by the Iceland mantle plume: New evidence from nitrogen elemental and isotope systematics of subglacial basalts. *Geochimica et Cosmochimica Acta* **176**, 206-226, doi:10.1016/j.gca.2015.12.021 (2016).

18 Mohapatra, R. K., Harrison, D., Ott, U., Gilmour, J. D. & Trieloff, M. Noble gas and nitrogen isotopic components in Oceanic Island Basalts. *Chemical Geology* **266**, 29-37, doi:https://doi.org/10.1016/j.chemgeo.2009.03.022 (2009).

19 Marty, B. & Humbert, F. Nitrogen and argon isotopes in oceanic basalts. *Earth and Planetary Science Letters* **152**, 101-112 (1997).

20 Thomazo, C. & Papineau, D. J. E. Biogeochemical cycling of nitrogen on the early Earth. *Elements* **9**, 345-351 (2013).

21 Grady, M. M., Wright, I., Carr, L. & Pillinger, C. Compositional differences in enstatite chondrites based on carbon and nitrogen stable isotope measurements. *Geochimica et Cosmochimica Acta* **50**, 2799-2813 (1986).

22 Thiemens, M. H. & Clayton, R. N. Nitrogen contents and isotopic ratios of clasts from the enstatite chondrite Abee. *Earth and Planetary Science Letters* **62**, 165-168, doi:https://doi.org/10.1016/0012-821X(83)90080-8 (1983).

23 Alexander, C. M. *et al.* The provenances of asteroids, and their contributions to the volatile inventories of the terrestrial planets. *Science* **337**, 721-723, doi:10.1126/science.1223474 (2012).

24 Hashizume, K. & Sugiura, N. Nitrogen isotopes in bulk ordinary chondrites. *Geochimica et Cosmochimica Acta* **59**, 4057-4069, doi:https://doi.org/10.1016/0016-7037(95)00236-S (1995).

25 Kung, C.-C. & Clayton, R. N. Nitrogen abundances and isotopic compositions in stony meteorites. *Earth and Planetary Science Letters* **38**, 421-435, doi:https://doi.org/10.1016/0012-821X(78)90117-6 (1978).

26 Sugiura, N., Kiyota, K. & Hashizume, K. Nitrogen components in primitive ordinary chondrites. *Meteorit Planet Sci* **33**, 463-482, doi:10.1111/j.1945-5100.1998.tb01651.x (1998).

27 Gibson, E. K., Moore, C. B. & Lewis, C. F. Total nitrogen and carbon abundances in carbonaceous chondrites. *Geochimica et Cosmochimica Acta* **35**, 599-604, doi:https://doi.org/10.1016/0016-7037(71)90089-5 (1971).

28 Grady, M. M. & Wright, I. P. Elemental and isotopic abundances of carbon and nitrogen in meteorites. *Space Science Reviews* **106**, 231-248 (2003).

29 Kerridge, J. F. Carbon, hydrogen and nitrogen in carbonaceous chondrites: Abundances and isotopic compositions in bulk samples. *Geochimica et Cosmochimica Acta* **49**, 1707-1714, doi:https://doi.org/10.1016/0016-7037(85)90141-3 (1985).

30 Pearson, V., Sephton, M., Franchi, I., Gibson, J. & Gilmour, I. Carbon and nitrogen in carbonaceous chondrites: Elemental abundances and stable isotopic compositions. *Meteorit Planet Sci* **41**, 1899-1918 (2006).

31 Robert, F. & Epstein, S. The concentration and isotopic composition of hydrogen, carbon and

nitrogen in carbonaceous meteorites. *Geochimica et Cosmochimica Acta* **46**, 81-95, doi:https://doi.org/10.1016/0016-7037(82)90293-9 (1982).

32 Sephton, M. A. *et al.* Investigating the variations in carbon and nitrogen isotopes in carbonaceous chondrites. *Geochimica et Cosmochimica Acta* **67**, 2093-2108, doi:https://doi.org/10.1016/S0016-7037(02)01320-0 (2003).

33 Ivanova, M. A. *et al.* The Isheyevo meteorite: Mineralogy, petrology, bulk chemistry, oxygen, nitrogen, carbon isotopic compositions, and 40Ar-39Ar ages. *Meteorit Planet Sci* **43**, 915-940, doi:10.1111/j.1945-5100.2008.tb01090.x (2008).
