# Supplementary material for: Nitrogen isotope evidence for Earth’s heterogeneous accretion of volatiles
Source: Nat Commun. 2022 Aug 15;13:4769. doi: 10.1038/s41467-022-32516-5 (PMC9378614; doi:10.1038/s41467-022-32516-5)
Supplement: Supplementary file 2 — Description of Additional Supplementary Files [file 41467_2022_32516_MOESM2_ESM.pdf]

Description of Additional Supplementary Files

File name: Supplementary Data 1

Description: Major element compositions of the starting silicates (in wt.%).

File name: Supplementary Data 2

Description: Summary of experimental conditions and products.

File name: Supplementary Data 3

Description: Major element compositions (in wt.%), N contents (in ppm), and $\delta^{15}N$ (‰) in silicate melts.

File name: Supplementary Data 4

Description: Major element compositions (in wt.%), N contents (in ppm), and $\delta^{15}N$ (‰) in metallic melts.

File name: Supplementary Data 5

Description: The modeled N-C-H-S contents and N-isotopes in Earth's different reservoirs.

File name: Supplementary Data 6

Description: The contribution of different types of impactors to Earth's major volatiles.
